# The application of procyanidins in diabetes and its complications: a review of preclinical studies

**DOI:** 10.3389/fphar.2025.1532246

**Published:** 2025-02-10

**Authors:** Yongchuang Zhang, Mengna Li, Haoyuan Liu, Yongfu Fan, Huan Huan Liu

**Affiliations:** ^1^ Department of Rehabilitation Medicine, The First Affiliated Hospital of Zhengzhou University, Zhengzhou, China; ^2^ Institute of Pain Medicine and Special Environmental Medicine, Nantong University, Nantong, China; ^3^ Rehabilitation Department, Henan Provincial Hospital of Traditional Chinese Medicine, Zhengzhou, China; ^4^ School of Rehabilitation Medicine, Henan University of Chinese Medicine, Zhengzhou, China; ^5^ International institute for Traditional Chinese Medicine, Guanzhou University of Chinese Medicine, Guangzhou, China

**Keywords:** diabetes mellitus, procyanidins, antioxidant, anti-inflammatory, insulin sensitivity

## Abstract

Diabetes mellitus (DM) and its various complications, including diabetic nephropathy, retinopathy, neuropathy, cardiovascular disease, and ulcers, pose significant challenges to global health. This review investigates the potential of procyanidins (PCs), a natural polyphenolic compound, in preventing and managing diabetes and its complications. PCs, recognized for their strong antioxidant, anti-inflammatory, and anti-hyperglycemic properties, play a crucial role in reducing oxidative stress and enhancing endothelial function, which are essential for managing diabetic complications. This review elucidates the molecular mechanisms by which PCs improve insulin sensitivity and endothelial health, thereby providing protection against the various complications of diabetes. The comprehensive analysis underscores the promising therapeutic role of PCs in diabetes care, indicating the need for further clinical studies to confirm and leverage their potential in comprehensive diabetes management strategies.

## Graphical Abstract

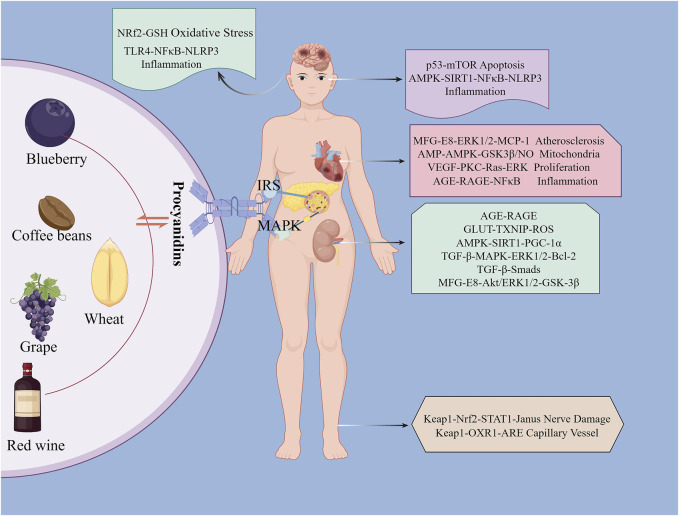

## 1 Introduction

**FIGURE 1:**
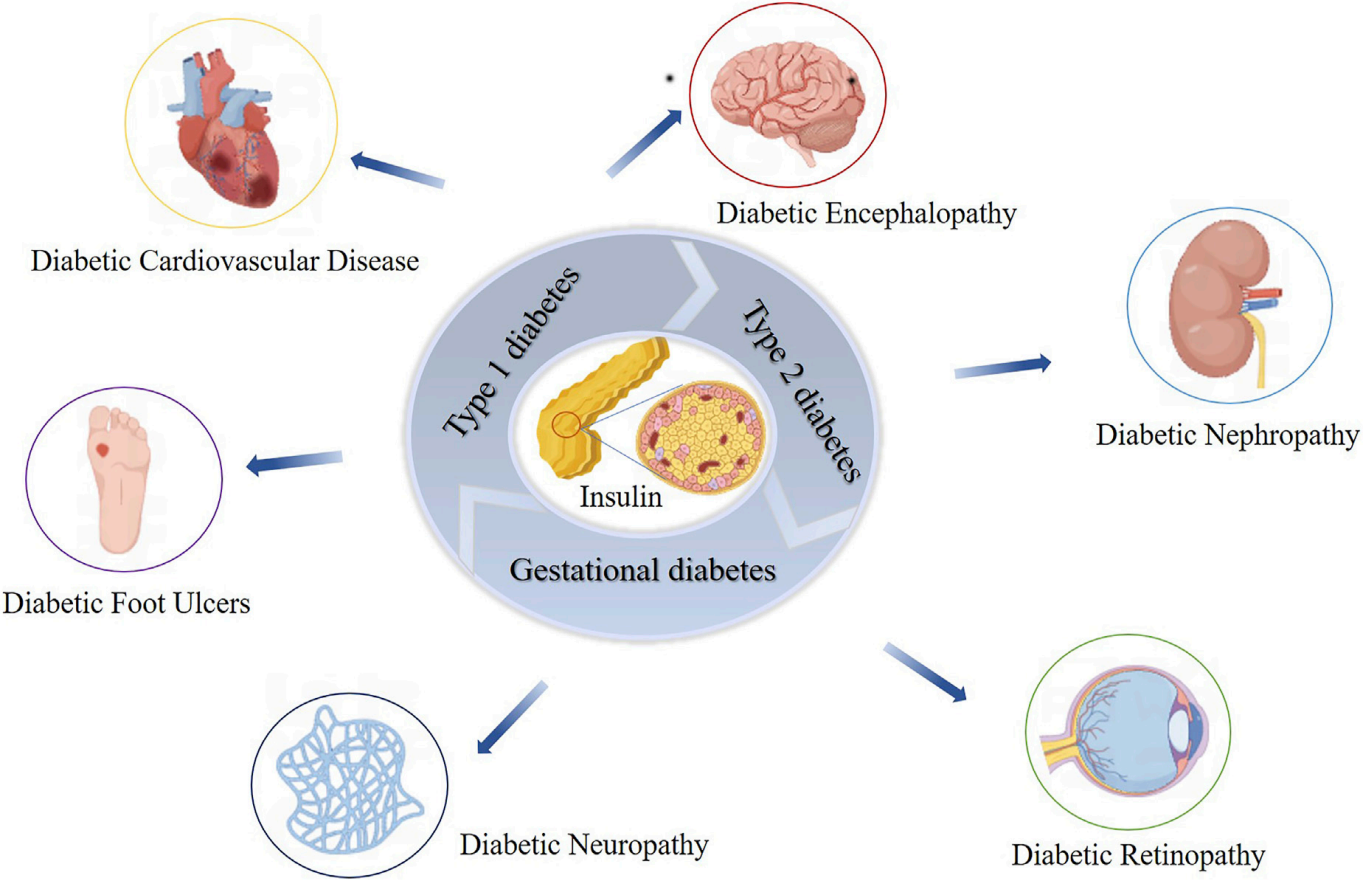
Impact of Insulin Dysfunction on Diabetes Mellitus and Related Complications. This figure illustrates the impact of insulin dysfunction on the development of diabetes mellitus and its associated complications. It shows how impaired insulin action leads to widespread metabolic disturbances, culminating in serious health issues such as nephropathy, retinopathy, neuropathy, cardiovascular disease, and diabetic ulcers.

DM is a major global health issue characterized by persistent hyperglycemia due to insufficient insulin secretion or action (Ill‐Min et al., 2020; Constanze et al., 2017; Zhimei et al., 2019). The main symptoms include increased appetite, thirst, urination, and weight loss (Diagnosis and Classification of Diabetes Mellitus, 2013). In 2021, approximately 536.6 million people aged 20–79 were diagnosed with diabetes globally, and this number is projected to reach 783.2 million by 2045 (Hong et al., 2022). Diabetes is triggered by genetic and environmental factors, leading to the autoimmune destruction of pancreatic-β-cells, impairing insulin production, and disrupting metabolic processes, resulting in hyperglycemia (Décio et al., 2020; Aleksey and Peter, 2008; Kathryn et al., 2013). Prolonged hyperglycemia can lead to severe complications such as diabetic nephropathy, neuropathy, retinopathy, and cardiomyopathy (Janika et al., 2022; David et al., 2023) (Figure 1). Cardiovascular diseases are the leading cause of death among diabetic patients, particularly cardiac complications (Justin et al., 2016; Anoop et al., 2015).

Over the past few decades, several pharmacological agents have been developed to regulate blood glucose. Metformin primarily reduces hepatic gluconeogenesis through the activation of AMP-activated protein kinase (AMPK), while sulfonylureas (e.g., glimepiride) enhance insulin secretion by closing pancreatic β-cell ATP-sensitive potassium channels (Reuben et al., 2005; Gaochao et al., 2001; Mary, 2004). Thiazolidinediones (e.g., pioglitazone) improve insulin sensitivity via peroxisome proliferator-activated receptor gamma (PPAR-γ) modulation (Aya et al., 2020; Zhiwei et al., 2021). More recent approaches include dipeptidyl peptidase-4 (DPP-4) inhibitors, which prolong the half-life of incretin hormones, and glucagon-like peptide-1 (GLP-1) receptor agonists, which stimulate insulin secretion and reduce glucagon levels (Daniel and Michael, 2006). Additionally, sodium-glucose cotransporter 2 (SGLT2) inhibitors lower blood glucose by promoting urinary glucose excretion (André, 2019). Current antidiabetic drugs mainly control blood glucose levels but cannot prevent or reverse complications (Dandan et al., 2020). Therefore, effective therapeutic strategies are urgently needed to halt or mitigate the progression of diabetes and its complications.

**FIGURE 2:**
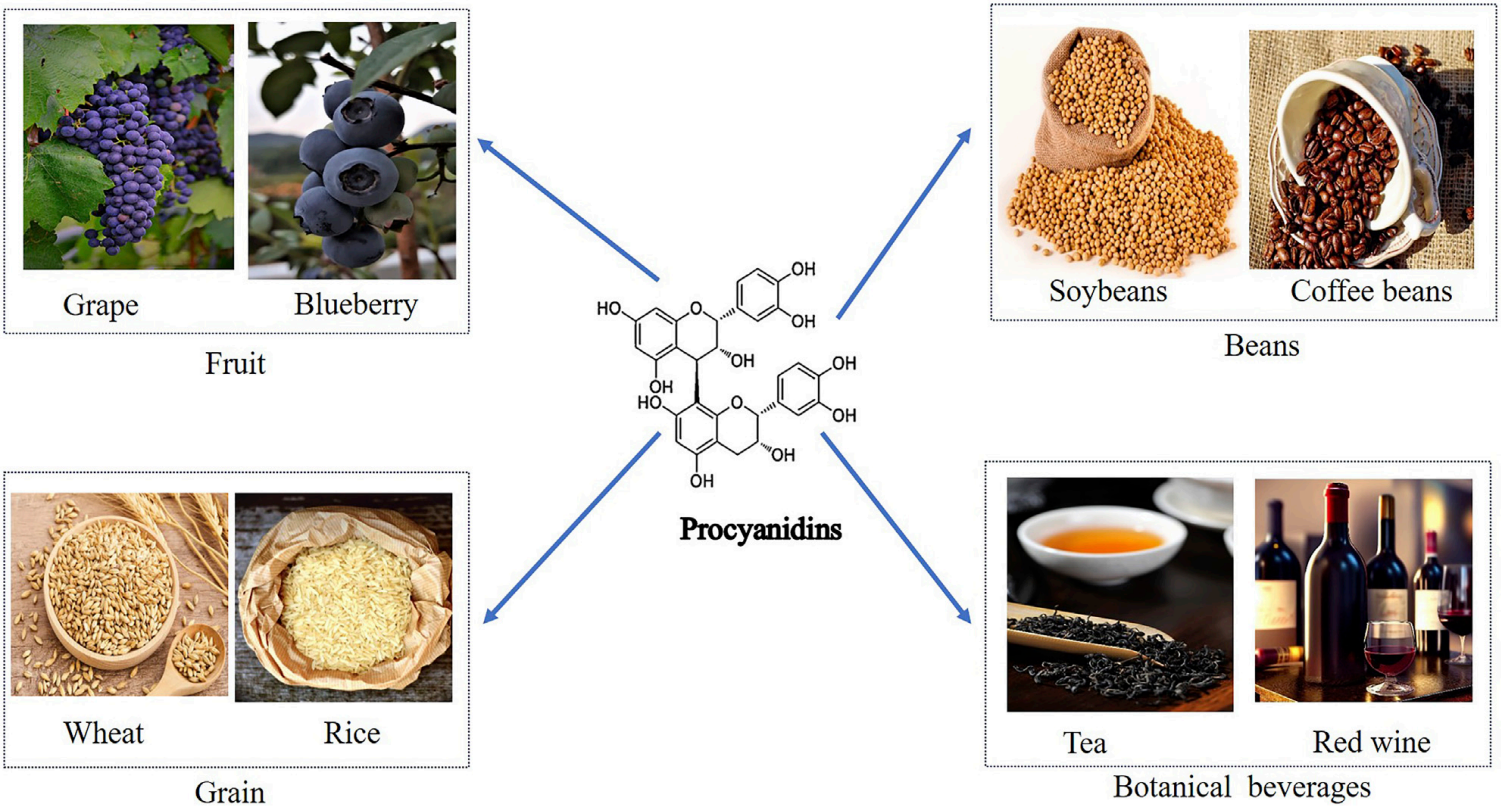
Dietary Sources of Procyanidins and Its Chemical Structure. This figure displays the major dietary sources of PCs and its chemical structure. The sources are grouped into categories including fruits like grapes and blueberries, beans such as soybeans and coffee beans, grains like wheat and rice, and botanical beverages including tea and red wine. The centralfigure illustrates the molecular structure of PCs, emphasizing its presence across diverse food groups.

PCs, composed of flavan-3-ol and flavan-3, 4-diol units, include catechins, apocynin, and gallocatechins (Chunhui, 2014; Liwei et al., 2002; Linards et al., 2022; Liang et al., 2016). Their main sources encompass grains, legumes, fruits, chocolate, and drinks like tea and wine (Abdur et al., 2019; Feng et al., 2016) (Figure 2). PCs exhibit diverse bioactivities such as anti-obesity, anti-diabetic, anti-cancer, anti-inflammatory, antioxidant, and cardiovascular protective effects (Eskandar et al., 2023; Antoni et al., 2012; Qian et al., 2023; Chuntang et al., 2022; Van Long et al., 2014; Liang et al., 2012). Studies suggest that PCs can lower blood glucose, improve insulin resistance, regulate insulin secretion, protect pancreatic β-cells in diabetic patients, and effectively alleviate diabetes complications (Javier et al., 2018; Suzanne et al., 2017; Anna et al., 2012). This review summarizes the molecular mechanisms and protective effects of PCs in diabetes treatment, which are shown in Table 1.

**TABLE 1:** Characteristics of the included studies.

Study	Animal/cell model	Groups	Dosage of PC	Administration method of PC	Intervation time	Beneficial effects and involved mechanisms
Castell-Auví et al. (2013)	Wistar rats	A. Group A Female (150–175) B. Group B male (130–150) C.Group C (225–250) D.Group D (175–200)	0/2.5/5/10/25/50 mg/kg/1,000 mg/kg	GSPE was gavaged for 36/21/45 days/1 h.	0–36 0–21 0–45 0–1 h	Low doses of procyanidins increased the plasma insulin levels and inhibited insulin gene expression, which led to reduced Pdx1 mRNA levels in the pancreas and reduced hepatic Ide gene expression
Caimari et al.	Wistar rats	A. VEH group:low-fat condensed milk B. GSPE group: 100 mg kg^−1^.day^−1^ of GSPE dissolved in low-fat condensed milk	100 mg kg^−1.^day^−1^ GSPE.	PC was oraled daily for 21 days after modelin	1-21 d	GSPE decreased levels of the downstream post-receptor target of adiponectin, p-AMPK, in the soleus muscle
Castell-Auví et al. (2013)	Wistar lean rats Zucker obese rat	Zucker rats:A. control group (Lean) B. Zucker obese group C. Zucker obese + GSPE group Wistar rats:A.control group B. GSPE group (25 mg GSPE/kg bw)	Zucker rats:35 mg GSPE/kg/day Wistar rats:25 mg GSPE/kg/day	Zucker rats:PC was oraled daily for 60 and 45 days after modelin	Zucker rats:0-60d Wistar rats:0-45d	GSPE targets 11b-HSD1 gene expression that dantidiabetic effects
Najim et al. (2004)	Sprague-Dawley rats	A.control group B.fructose-enriched diet group C.anthocyan-treated fructose-fed group D.procyanidin and galloylated procyanidins-treated fructose-fed group E.vitaflavan-treated fructose-fed	21.42 mg/kg of total polyphenols, 10 mL/Kg	Polyphenols was gavaged for 6 weeks	0–6 weeks	Polyphenolic treatments prevented the increased expression of the p91phox NADPH oxidase subunit
Tsai et al.	Sprague-Dawley rats	A.control group (standard Purina chow) B. group F (60% HF Diet) C. group L (HF + PC 125 mg/kg) D. group H (HF + PC 250 mg/kg)	Low-dose group: PC (125 mg dissolved in 0.1 g PC/mL H2O)/kg High-dose group: PC (250 mg (dissolved in 0.2 g PC/mL H2O)/kg	PC was gavaged for 14 weeks	0–14 weeks	PC are associated with amelioration of defective insulin action on specific postreceptor insulin signaling related to IRS-1 and GLUT 4 proteins expression
Khanal et al.	Sprague-Dawley rats	A. Control group B. HF group C. HF group + LPC D.HF group + MPC E.HF group + HPC	Low-dose group: 3.3 g/kg; Medium dose group: 6.6 g/kg) High-dose group: 33 g/kg	PC was oraled daily for 7 weeks	2–7 weeks	PC in the medium level at 6.6 g/kg diet was the most effective in improving factors associated with metabolic syndrome in the high fructose fed growing rats sed in the current study
Liu et al.	C57BL/6 mice	A. Model group B. positive control group C. Low-dose PC D. Medium-dose PC E. High-dose PC	Low-dose PC: 75 mg/kg; Medium-dose PC: 150 mg/kg High-dose PC: 300 mg/kg	PC was oraled daily for 4 weeks	0–4 weeks	PC ameliorated insulin resistance by decreasing LPS/TLR4/JNK inflammatory response, and enhancing IRS1/PI3K/AKT insulin signaling pathways in the liver
Cordero-Herrera et al.	Human HepG2 cells	A.EC group B.CPC group	10 μM or1 μg/mL	HepG2 incubated were incubated with 10 μM or 1 μg/mL PC for 24 h	0–24 h	EC and CPE pre-treatment also prevented the inactivation of the PI3K/AKT pathway and AMPK, as well as the diminution of GLUT-2 levels induced by high glucose
Rui et al. (2020)	HepG/HT22	A. Normal group B. Model group C. Positive group D. Treatment group	3.75 μg/mL LSF	HepG2/HT22 were incubated with 3.75 μg/mL LSF for 24 h	0–24 h	Catechin, procyanidin A1, and procyanidin A2 are the main components in LSF that inhibit Tau hyperphosphorylation through improving IR via the IRS-1/PI3K/Akt/GSK-3β pathway
Gemma et al. (2010a)	Wistar female rats	A. Control group B. Cafeteria group C. Cafeteria+25 mg/kg D. Cafeteria+50 mg/kg	Low-dose:25 mg/kg/d GSPE High-dose:50 mg/kg/d GSPE	GSPE was oraled daily for 10 days (short treatment)/30 days (long treatment)	0-10d 0-30d	GSPE is dependent on Irs1 when stimulating glucose uptake but also suggests that GSPE has a direct effect on Glut4 transporter activity
Xinyi et al. (2020)	ApoE KO mice	A.control group B. ApoE KO group C. ApoE KO + PC	100 mg/kg/d	PC was oraled daily for 24 weeks	0–24 weeks	PC could ameliorate atherosclerosis in ApoE-KO mice by improving NO bioavailability and reducing oxidative stress through NADPH oxidaseJdependent mechanisms
Wang et al.	C57BKS db/db	A. vehicle (saline) B. CD-1 (20 mg/kg) C. CD-1 (20 mg/kg)+CQ (20 mg/kg)	20 mg/kg	CD-1 were daily administrated (intraperitoneal injection) with 20 mg/kg for 5weeks	0–5 weeks	CD-1 on activation of Keap1/Nrf2 antioxidant signaling pathway and the amelioration of inflammation, endoplasmic reticulum stress, andapoptosis were through autophagy
Yuta et al. (2013)	KK-A^y^ mice	A. Control B. BE diet	22.0 g of BE/kg	PC was oraled daily for 1 week	0–1 week	PC via the activation of AMP-activated protein kinase (AMPK) reduced blood glucose levels and enhanced insulin sensitivity
Rodríguez-Carrizalez et al. (2014)	Wistar rats	A. Control group B. GSPE group	25 mg/kg bw/d	GSPE was gavaged for 45 days	0-45d	Procyanidin inhibition of intestinal DPP4 activity, either directly and/or via gene expression downregulation, could be responsible for some of their effects in glucose homeostasis.
Bak et al.	RAW 264.7 cells	A.Control B.5ug/mL C.10 ug/mL D.25 ug/mL E.30ug/mL F.50ug/mL	5ug/mL/10 ug/mL/25 ug/mL/30ug/mL、50ug/mL	Cells were treated with the indicated concentration of WGP for 24 h	0–24 h	WGP exerts potent anti-inflammatory activity through the inhibition of iNOS and COX-2 by regulating NFκB and p38 MAPK pathway
Liu and Wu. (2021)	C57BL/6 mice	A. Chow group B. HFHS group C. HFHS + PC group D. Abs group	27.8 mg/kg bw	PC was gavaged for 4 weeks	0–4 weeks	PA improved IR via NF-κB/NLRP3 pathway in GDM and *postpartum* mice, which partly through its metabolites by gut microbiome
Tian et al.	Sprague Dawley rats	A.DMSO B.PC	PCB2 (0.1–10 μM)	PCB2 (0.1–10 μM) for 24 h	24 h	PCB2 regulated macrophage M2 polarization via the activation of PPARγ
Farid et al.	MSCs cell	A. gp I: healthy control B. untreated induced type I diabetic group C. gp III: GSE-treated diabetic group D. gp IV: MSCs-treated diabetic group E. gp V: GSE- and MSCs-treated diabetic group	300 mg/kg	PCB1/PC1 was oraled daily for 30 days	0–30 days	PCB1/C1/MSCs therapy in type I-induced diabetic rats has dramatically managed homeostasis of glucose and insulin secretion; together with, improvement in levels of inflammatory markers and oxidative stress
Tie et al.	ICR male	A. normal control group B. model group C. Fen group D. PC-H group E. PC-L group	200 mg/kg/50 mg/kg	PC was oraled daily for 16 weeks	0–16 weeks	Proanthocyanidins ameliorated lipid metabolism and attenuated hepatic steatosis in mice with HFD/STZ-induced T2DM, for which activation of AMPK/ACC/CPT1A signaling might be an underlying mechanism
Nie et al. (2020)	HUVECs cell	A.Vehicle B.HG C.HG + PCB2	10 μM	HUVECs were incubated with 10 μM PCB2 for 24 h	24 h	PCB2 on ER stress and endothelial dysfunction required the inter-dependent actions of PPARδ and AMPK.
Muthenna et al. (2014)	Wistar rats	A. control B. Diabetic(D) C. D + cinnamon D. D + PCB2	0.002% PCB2	Type 2 diabetes 3% cinnamon or 0.002% PCB2 fed in diet for 12 weeks	0–12 weeks	PCB2 suppressed renal AGE-RAGE stimulated MCP-1 and PKC-a expression, thereby modulated slit diaphragm proteins nephrin and podocin expression
Zhang et al. (2013)	C57BLKS/J db/db	A.control (db/m) B. Vehicle (C57BLKS/J db/db, saline) C. Diabetic group (GSPB2 (30 mg/kg/day)	30 mg/kg/day	GSPB2 was oraled daily for 10 weeks	0–10 weeks	GSPB2 treatment significantly decreased protein levels of MFG-E8, phospho-ERK1/2, phospho-Akt, and phospho-GSK-3β in the kidneys of db/db mice
Li et al. (2015)	HK-2 cell	A.Normal group B.High glucose C. Normal group + PC D. High glucose + PC	10 µM PCB2	HK-2 cell was incubated with 10 μM PCB2 for 24 h	0–24 h	PCB2 inhibited of the expression of TGF-β, p-Smad2 and 3, by modulating P38/MAPK signaling pathway inhibited HG-induced EMT.
Cai et al. (2016)	Mouse podocyte clonal cells (MPC5)	A.Normal group B.High glucose C. High glucose + PCB2(0、0.625、1.25、2.5、5、10、20、40 μg mL^−1^)	0/0.625/1.25/2.5/5/10/20/40 μg mL^−1^	MPC5 cell was incubated with of GSPB2 (0/0.625/1.25/2.5/5/10/20/40 μg mL^−1^) in 30 mM glucose for 48 h	48 h	GSPB2 activated of AMPK-SIRT1-PGC-1α signalling, protected podocytes from high glucose-induced mitochondrial dysfunction, oxidative stress and apoptosis
Wei‐Jian et al. (2015)	C57BLKS/J db/db	A. db/m B. db/m + GSPE C. db/db D. db/db + GSPE	30 mg/kg	GSPE was given 30 mg/kg/day, gavage for 12 weeks	12 weeks	GSPE enhanced p38MAPK-ERK1/2 signaling pathway oxidase activity, suppressed renal cell apoptosis and expression of TXNIP in renal cell apoptosis
Zhou et al. (2016)	C57BLKS/J db/db	A. Vehicle B. GSPB2	30 mg/kg	GSPB2 was given 30 mg/kg/day, gavage for 10 weeks	10 weeks	GSPB2 by suppressing expression of nuclear factor-κB (NF-κB) p65 in nuclear extracts and restoring expression of Mimecan protein has beneficial effects on oxidative stress and renal fibrosis in the diabetic kidney
Li et al. (2022)	Retinal pigment epithelial cells (RPE)	A.Normal control group B. High glucose group C.High glucose with PC groups	PC (0.05/0.1/0.5/1/5/10/50/and 100 μM)	RPE cells were incubated with of GSPB2 (0.05/0.1/0.5/1/5/10/50/and 100 μM) in 30 mM glucose for 48 h	48 h	PC throughs the p53/mTOR autophagy pathway to protect RPE cells from high glucose-induced injury
Zou et al. (2023)	Rat retinal capillary endothelial cells (TR-iBRB2)	A.High glucose B.High glucose + PCB2	PCB2 (0/5/10 and 25 µM)	TR-iBRB2 cells were pre-treated with various concentrations of PCB2(0, 5, 10 and 25 µM) for 24	24 h	PCB2 Protects TR-iBRB2 Cells Against Hyperglycemia Stress by Attenuating Oxidative Stress and Inflammasome Activation via Regulation of Redoxosomes/NF-kB Signaling
Fan et al. (2021)	Endothelial progenitor cells (EPCs)	A. Mannitol B. HG C. HG + PCB2(0.1 μmol/L, 0.5 μmol/L, 2.5 μmol/L)	PCB2 (0.1 μmol/L, 0.5 μmol/L, 2.5 μmol/L)	EPCs were pre-treated with various concentrations of PCB2(0.1 μmol/L/0.5 μmol/L/2.5 μmol/L) for for 24 h	24 h	PCB2 treatment accelerates wound healing and increases angiogenesis in diabetic mice, which may be mediated by activating of Nrf2 improving the mobilization and function of EPCs
Li et al. (2013)	HUVEC	A. gly-LDL B. gly-LDL + GSPB2-L (2.5umol/L) C. gly-LDL + GSPB2-M(5.0umol/L) D. gly-LDL + GSPB2-H (10.0umol/L)	GSPB2(2.5umol/L/5.0umol/L/10.0umol/L)	HUVEC were pre-treated with various concentrations of GSPB2 (2.5, 5.0, 10.0 μmol/L) for 48 h	48 h	Procyanidin B2 (GSPB2) protect against gly-LDL induced VEC apoptosis through PIMT regulation
Yu et al. (2012)	C57BLKS/J db/db db/m mice	A. Control db/m group B. DMT:untreated db/db group C. DMT + GSPB2 group	GSPB2 (30 mg/kg/day)	GSPB2 was given diluted in normal saline solution by intragastric administration for 10 weeks	10 weeks	GSPB2 inhibits aortic expression and serum level of MFG-E8 and reduces atherogenesis in db/db mice
Luan et al. (2014)	C57BLKS/J db/db db/m mice	A. Control db/m group B. DMT:untreated db/db group C. DMT + GSPB2 group	GSPB2 (30 mg/kg/day)	GSPB2 was given diluted in normal saline solution by intragastric administration for 10weeks	10 weeks	GSPB2 activates S100A11/RAGE/PPAR γ signaling pathway to alleviate cardiac fibrosis in advanced glycation end-induced rats in type 2 diabetic rats

## 2 Pharmacokinetics of procyanidins

**FIGURE 3:**
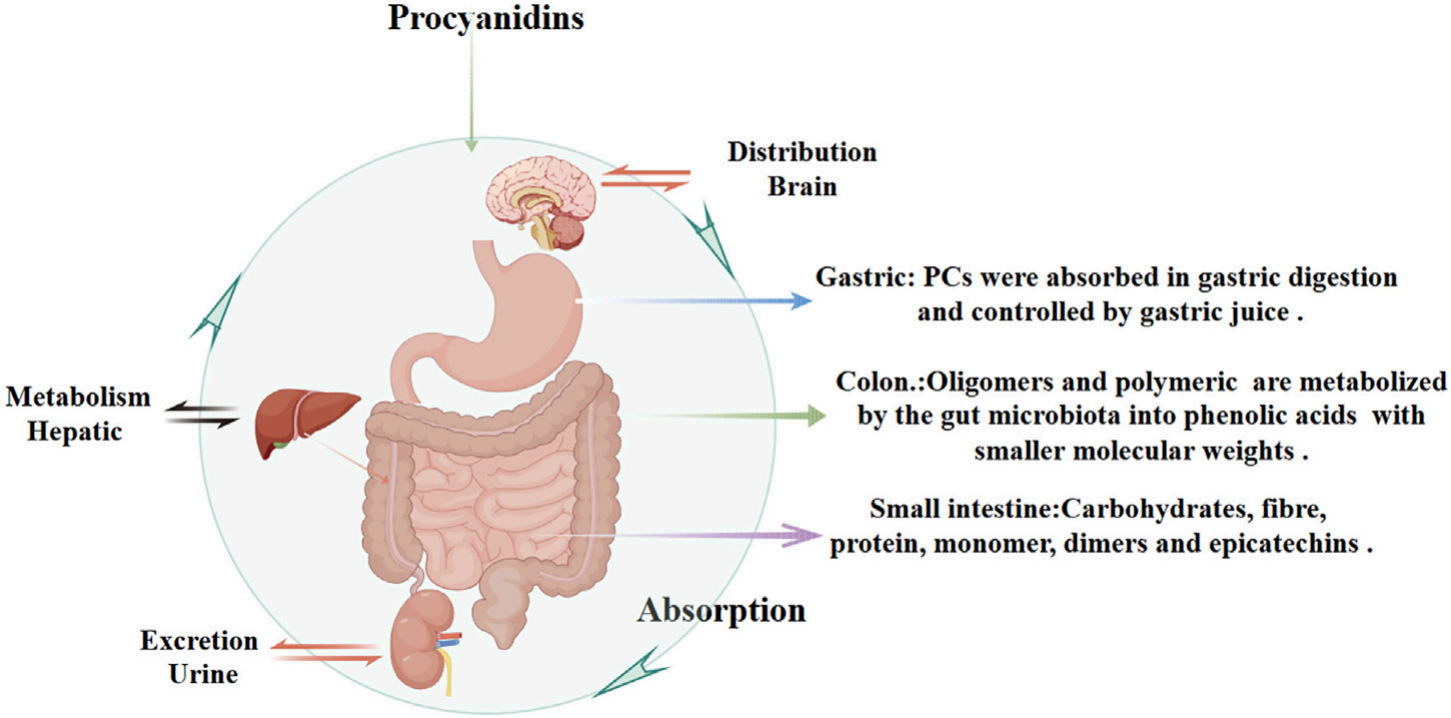
Pharmacokinetics of Procyanidins. This figure illustrates the absorption, distribution, metabolism, and excretion of PCs. PCs are partially absorbed in the stomach and small intestine, with interactions involving dietary components like carbohydrates, fibers, and proteins. In the colon, unabsorbed PCs are metabolized by gut microbiota into phenolic acids with smaller molecular weights, improving solubility and systemic absorption. Hepatic metabolism further processes PCs, and their metabolites are eventually excreted through urine.

PCs are characterized by their double and single bonds, with catechols or epicatechols as fundamental components (Kaul, 1996). The health benefits of PCs are constrained by their low oral bioavailability due to incomplete intestinal absorption and extensive metabolism and excretion (Brigitte et al., 1989; Yue et al., 2021; Claudine et al., 2005; Ying Yng et al., 2014). It is estimated that over 90% of dietary polyphenols remain in the colon, with only a small fraction being indirectly absorbed in the small intestine through interactions with carbohydrates, fibers, and proteins (Michael, 2004; Gary and Claudine, 2005). Multiple factors influence the hydrolytic absorption of PCs, including gastrointestinal pH, dietary composition, and solubility in gastric and intestinal fluids. The acidic environment of the stomach may facilitate initial PCs release from food matrices but can also degrade certain oligomers, reducing their structural integrity and bioavailability. In the small intestine, dietary fibers and proteins can form complexes with PCs, limiting absorption, whereas lipids may enhance PCs solubility through micelle or emulsion formation. Tannins can further decrease bioavailability by competing for binding sites or forming insoluble complexes. Once in the colon, unabsorbed PCs oligomers and polymers interact with enterocyte membrane proteins, potentially modulating metabolic pathways (Liang et al., 2016). Meanwhile, gut microbiota convert these higher-order PCs into smaller phenolic acids (e.g., hydroxybenzoic, hydroxyphenylacetic, and hydroxycinnamic acids) with better solubility and increased absorption (Maaike et al., 2009). Ultimately, these metabolites are excreted in the urine (Figure 3).

Low bioavailability remains a major hurdle for harnessing the therapeutic benefits of PCs, necessitating innovative delivery approaches such as nano-encapsulation, phospholipid complexation, and co-administration with permeability enhancers. These strategies aim to stabilize PCs structure against pH-induced degradation, improve solubility, and promote systemic circulation. Further studies are required to elucidate the optimal conditions, formulations, and dietary adjuncts that maximize PCs efficacy in diabetes management. By advancing our understanding of PCs pharmacokinetics, including absorption, distribution, metabolism, and excretion, and refining targeted formulations, we can better leverage their therapeutic potential in preventing and managing diabetes and its complications.

## 3 Methods

### 3.1 Literature search

**FIGURE 4:**
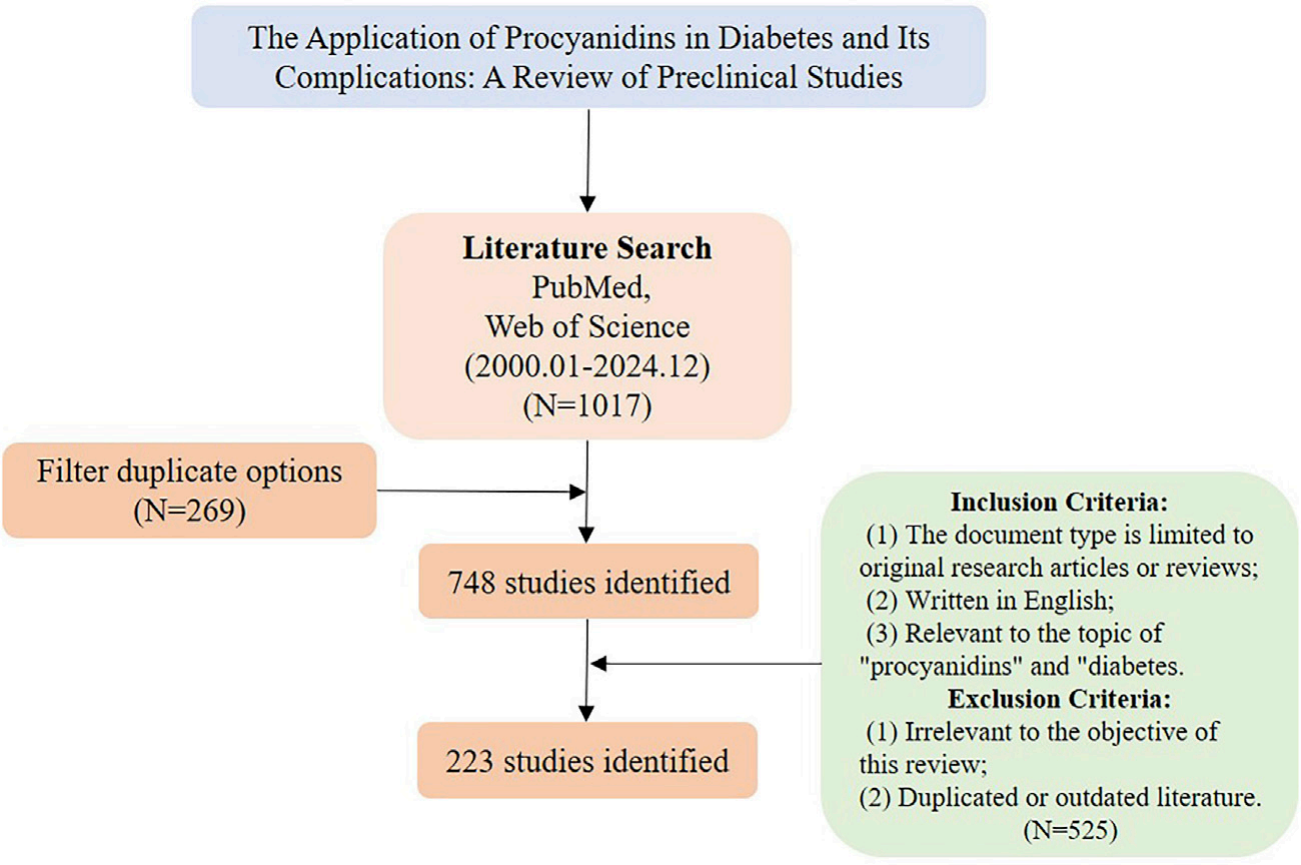
Literature screening process diagram.

We conducted a comprehensive search on the PubMed (www.pubmed.com) and Web of Science (www.webofscience.com) databases for articles on procyanidins and diabetes published from 1 January 2000, to 30 December 2024. The research methodology is illustrated in Figure 4.

### 3.2 Search strategy

Procyanidins OR Procyanidin B2 OR GSPE OR Procyanidin B3 OR Catechin OR flavan-3-ol) AND (Diabetes mellitus OR diabetic nephropathy OR diabetic retinopathy OR diabetic neuropathy OR diabetic foot ulcer OR diabetic encephalopathy OR diabetic cardiovascular disease OR diabetic cardiomyopathy.

### 3.3 Inclusion criteria

(1): The document type is limited to original research articles or reviews; (2) Relevant to the topic of “procyanidins” and “diabetes”.

### 3.4 Exclusion criteria

(1) Irrelevant to the objective of this review; (2) Duplicated or outdated literature.

## 4 Mechanism of the protective effect of PCs on diabetes mellitus

### 4.1 Improve the insulin resistance

Insulin, an essential hormone secreted by pancreatic β-cells, regulates glucose metabolism and exerts a profound influence on blood glucose levels (Gary and Patricia, 2021). Typically, insulin binds to receptors on the surfaces of target cells, thereby reducing hepatic glucose output and promoting glucose uptake in skeletal muscle and adipose tissue (Benedetta et al., 2019). When various internal and external factors drive target cells toward insulin resistance, greater amounts of insulin are required to trigger glucose transport and utilization. This phenomenon, referred to as insulin resistance, features elevated glucose and insulin levels in the bloodstream (Rosalyn and Solomon, 1960; Christian et al., 2013).

**FIGURE 5:**
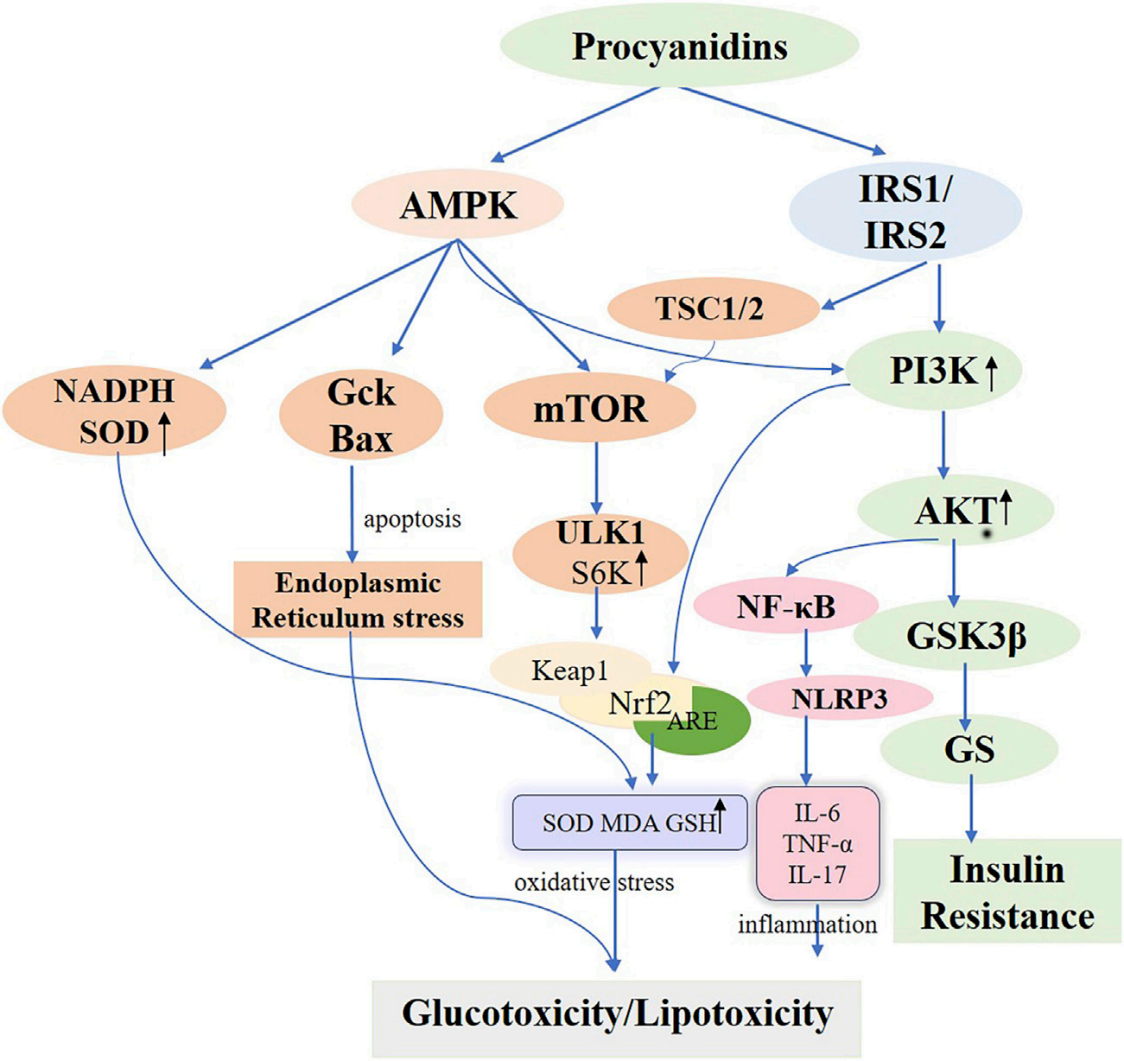
Molecular Mechanisms of Procyanidins in Modulating Diabetes Mellitus. This figure delineates the comprehensive molecular pathways impacted by PCs in the management of DM. PCs enhance insulin sensitivity by activating PI3K/AKT signaling via IRS1/IRS2 and reduce insulin resistance through GSK3β inhibition. PCs also alleviate oxidative stress by activating AMPK and Nrf2 pathways, increasing antioxidants like SOD and GSH, while reducing inflammatory responses mediated by NF-κB and NLRP3. Additionally, PCs modulate cellular stress and autophagy, mitigating endoplasmic reticulum stress and promoting metabolic homeostasis. These mechanisms collectively address glucotoxicity, lipotoxicity, and insulin resistance in diabetes.

Located in the cell membrane, the insulin receptor exhibits a tetrameric structure with two α-chains and two β-chains, serving as the primary hub for insulin action and orchestrating the insulin signaling cascade. Upon binding to the α-chain, insulin governs glucose transport and adiposity and modulates cell proliferation and differentiation. This interaction enhances gluconeogenesis while suppressing adipose tissue catabolism (Marinko et al., 2021). When insulin engages the β-chain, it triggers the insulin receptor substrate (IRS1/2), thereby boosting glucose metabolism (via the PI3K/PKB/AKT pathway), protein synthesis (via the TSC1/2-mTOR pathway), and cell proliferation and differentiation (via the RAS-MAPK-ERK1/2 pathway) (Rebecca et al., 2017; Barbara Di et al., 2016; Teri et al., 1991). Interruptions throughout the insulin signaling pathway can ultimately lead to insulin resistance. Often, diverse kinases and phosphatases—for instance, phosphorylated tyrosine—amplify serine/threonine phosphorylation, inactivating IRS proteins and worsening insulin resistance (Kyle and Morris, 2012). Additionally, processes involving kappa kinase β (IKK-β), c-Jun N-terminal kinase (JNK-1), protein kinase C (PKC), and mammalian target of rapamycin (mTOR)-driven serine modification of IRS1 also contribute to insulin resistance, further intensified by free fatty acids, lipotoxicity, oxidative stress, and inflammation (Stergios et al., 2009). PCs are frequently cited for their ability to improve insulin sensitivity in experimentally induced diabetic rats (Antoni et al., 2017; Anna et al., 2013). Previous studies underscore the marked antihyperglycemic effects of PCs in rats maintained on a high-fructose diet (Najim et al., 2004; Takako et al., 2008; Hsiang‐Jung et al., 2008). Ramesh CK and colleagues found that PCs extracts from cranberries and blueberries, administered prophylactically, significantly mitigated insulin resistance, boosted glucose sensitivity, and lowered blood glucose in diabetic rats fed a high-fructose diet, reinforcing the therapeutic value of PCs (Ramesh et al., 2010; Ramesh et al., 2012). M Liu et al. provided PCs to T2D mice (75, 150, and 300 mg/kg) via continuous gavage for 4 weeks, documenting improvements in intestinal barrier integrity by restoring morphology and raising the expression of tight junction proteins, including Zonula occludens-1 (ZO-1), Claudin-1, and occludin. This approach substantially reduced insulin resistance, promoted glucose uptake, and lowered blood glucose levels by activating the IRS1/PI3K/AKT signaling pathway (Min et al., 2022). Further *in vitro* research corroborated these results. I Cordero-Herrera et al. cultured insulin-sensitive human HepG2 cells under high-glucose conditions and administered cocoa-derived polyphenols and epicatechin, which blocked tyrosine phosphorylation and lowered total IR, IRS-1, and IRS-2 levels through PI3K/AKT and AMPK pathway inhibition, thereby mitigating high-glucose-induced insulin signaling impairment that alters gluconeogenesis (Isabel et al., 2014). The activation and subsequent engagement of the insulin receptor (IR) and its downstream mediators, IRS-1 and IRS-2, remain critical for regulating insulin signaling (Mary et al., 2002). In hepatic insulin resistance, normal tyrosine phosphorylation of IR and IRS is compromised, diminishing PI3K activity associated with IRS. Conversely, the oligomeric form of grape seed procyanidin (GSPE) extract induces tyrosine phosphorylation, thereby activating the IR and decreasing AKT and GSK-3β phosphorylation via PI3K/AKT pathway stimulation. This process augments GS phosphorylation, thereby improving hepatic insulin sensitivity (Aurèle et al., 2019; Rui et al., 2020; Gemma et al., 2010a) (Figure 5). Meanwhile, a glucose-lowering formulation containing hawthorn polyphenols, D-chiro-inositol, and epigallocatechin gallate exhibited synergistic hypoglycemic activity, markedly improving insulin resistance in mice, decreasing fasting blood glucose and hepatic gluconeogenesis, and enhancing hepatic glycogen synthesis and storage (Chao et al., 2021). Additionally, a randomized controlled trial revealed that a combination of flavan-3-ols and isoflavones enhanced insulin sensitivity and lipoprotein profiles compared to placebo, further lowering the estimated 10-year CVD risk in women with type 2 diabetes (Peter et al., 2012).

In summary, under insulin-resistant conditions, polyphenolic PCs can effectively bolster cellular insulin sensitivity. However, prior investigations have yet to establish the ideal PCs concentration and dose for mitigating insulin resistance or to clarify the therapeutic distinctions among active metabolites from diverse sources. Future studies should center on identifying effective concentrations and characterizing botanical metabolites derived from multiple sources.

### 4.2 Reduced glucotoxicity and lipotoxicity

Glucotoxicity is characterized by irreversible tissue and cell damage resulting from insulin resistance and persistently elevated glucose levels stemming from excessive carbohydrate intake (Mota et al., 2016). The primary cause of glucotoxicity is insulin resistance, which arises from defective islet cell function and damage caused by reduced insulin mRNA expression, associated with diminished transcription or activity of transcription factors involved in insulin production (Daniël and Michaëla, 2011). Insulin resistance significantly contributes to hyperglycemia-induced glucotoxicity. Excess carbohydrates are metabolized into free fatty acids and triglycerides, promoting adipogenesis and lipoatrophy through the activation of enzymes like acetyl coenzyme A carboxylase (ACC), fatty acid synthase (FAS), and SCD-1 (Mota et al., 2016). These processes are influenced by endoplasmic reticulum (ER) stress, oxidative stress, mitochondrial dysfunction, and islet inflammation (Maria et al., 2020; Wenqian et al., 2018; Jiwon and Kun‐Ho, 2011).

Prolonged exposure to elevated fatty acid levels triggers lipotoxicity, compromising pancreatic β-cell functionality and viability. This condition activates multiple stress pathways, resulting in β-cell impairment and mortality. ER stress and oxidative stress are especially well-documented contributors to this effect (Kerry et al., 2005; Eloisa Aparecida et al., 2021; Andrei et al., 2007). Cellular lipotoxicity, also known as glycolipotoxicity, is exacerbated by elevated glucose levels (Yiqi et al., 2022). Nicotinamide adenine dinucleotide phosphate (NADPH) oxidase (NOX), a producer of reactive oxygen species (ROS), acts as a key generator of oxidative stress within pancreatic islet β-cells, significantly affecting glucose-induced insulin release and β-cell viability in Type 1 and Type 2 diabetes (Eloisa Aparecida et al., 2021). Animal studies demonstrate that palmitate impairs insulin secretion by upregulating the p47phox NOX subunit protein within rat pancreatic islets (Marta et al., 2010). Shuang et al. (2017) demonstrated that PCs from lychee pericarp decrease mRNA levels of cellular NADPH oxidase subunits (p47phox, p67phox, NOXJ2/gp91phox, and NOXJ4) and enhance superoxide dismutase (SOD) activity, thereby providing a strong antioxidant effect. Additionally, Xinyi et al. (2020) found that cinnamon tannin D1, an A-type PCs oligomer derived from Cinnamomum cinnamomi, protects pancreatic β-cells against glucotoxicity induced by high glucose and palmitic acid, both *in vitro* and *in vivo*. The key process entails CD-1 initiating autophagy in pancreatic β-cells via the AMPK/mTOR/UNC-52-like kinase 1 (ULK1) pathway. This autophagic response reduces cell damage by activating the Keap1/Nrf2 antioxidant pathway, which is associated with Kelch-like ECH protein 1 and nuclear factor erythroid 2-related factor 2, thereby mitigating inflammation, endoplasmic reticulum stress, and apoptosis. Consequently, this lowers hyperglycemia and reduces glycotoxicity in diabetic db/db mice (Figure 5). A clinical study found (Hadi et al., 2020) that supplementation with epigallocatechin-3-gallate significantly increased serum total antioxidant capacity (TAC) levels in patients with type 2 diabetes, thereby enhancing the body’s overall antioxidant capacity.

In summary, this section systematically elucidates the mechanisms through which glucotoxicity and lipotoxicity impact pancreatic β-cell function and survival, leveraging both literature and experimental evidence to underscore the potential application of PCs in mitigating these pathological processes. In particular, PCs extracted from lychee pericarp and A-type PCs oligomers (CD-1) derived from cinnamon exhibit promising effects on autophagy activation, mitigation of β-cell damage, and enhancement of glucose metabolism. However, a notable gap remains between these findings and their clinical application. First, many uncertainties exist regarding the absorption, distribution, and metabolism of PCs in humans, including their bioavailability and potential side effects. Moreover, given the trend towards precision medicine, which accounts for individual differences, factors such as a patient’s disease stage, comorbidities, and genetic background must also be considered to fully assess the clinical translational potential of these active compounds in diabetes management. Only by bridging laboratory research with evidence-based medicine can we fully harness the potential of these active components in suppressing glucolipotoxicity and reducing the risk of diabetic complications.

### 4.3 Maintains glucose homeostasis

Maintaining glucose homeostasis is crucial for providing energy to vital organs and sustaining overall health. The liver is key in this dynamic, participating in processes such as glucose synthesis, glycogenolysis, glycolysis, gluconeogenesis, and glucose metabolism. Insulin, primarily stimulated by glucose, is secreted by beta cells under normal physiological conditions. During fasting, the liver generates glucose to fuel the brain, muscles, and adipose tissues, collectively regulating glucose uptake (Xinyi et al., 2020; Wei et al., 2017). PCs regulate glucose uptake, effectively lowering glucose levels through this mechanism (Montserrat et al., 2012). Recent research has underscored the importance of AMPK in managing metabolic conditions, including diabetes, obesity, and fatty liver disease (Sébastien and Reuben, 2017). Furthermore, AMPK boosts glucose absorption in insulin-responsive tissues such as adipocytes, muscle cells, and hepatocytes, proving effective in enhancing glucose uptake *in vitro* (Zhang et al., 2009; Yuta et al., 2013; Montserrat et al., 2004; Isabel et al., 2015). Elevated phosphorylation levels of AMPK were noted in the liver, muscle tissue, and adipocytes of mice subjected to insulin resistance from high-fat diets and streptozotocin (STZ)-induced diabetes models (Masahito et al., 2010; Peiling et al., 2013). Yuta et al. (2013), Ken-yu et al. (2022) utilized anthocyanins extracted from black beans to treat type 2 diabetic mice, activating AMPK in skeletal muscle cells to improve glucose utilization and decrease gluconeogenesis, while concurrently down-regulating phosphoenolpyruvate carboxykinase (PEPCK) and glucokinase (GK) activities—crucial enzymes in glycolysis that boost intracellular glucose catabolism.

Glucose Transporter Type 4 (GLUT4), a crucial glucose transport regulator, is predominantly found in adipose, skeletal, and heart muscles. Significantly, skeletal muscle serves as a crucial target for managing hyperglycemia, accounting for roughly 80% of postprandial insulin-mediated glucose absorption and is essential for maintaining glucose equilibrium (Nia et al., 2002; Alan and Kahn, 2001). PCs enhance glucose absorption, facilitate GLUT4 translocation in mouse L6 myotubes, and promote glucose uptake within skeletal muscle via insulin and AMPK-dependent pathways (Miki et al., 2011). G Montagut et al. and HH Lee et al. showed that enhanced GLUT4 translocation increases Akt phosphorylation in adipocytes, further boosting a critical pathway in the insulin signaling cascade (Gemma et al., 2010b; Hee-Hyun et al., 2010). Additionally, PCs affect glucose synthesis and metabolism by boosting the functions of enzymes including glucokinase, hexokinase, and glycogen synthase, and reducing the functions of liver enzymes like glucose-6-phosphatase, phosphoenolpyruvate carboxykinase, and fructose-1,6-bisphosphatase (Zhang et al., 2009; Gopalsamy Rajiv et al., 2011) (Figure 5).

GLP-1, a crucial hormone linked to intestinal insulin, is secreted by L-cells within the intestinal lining and remains vital for human glucose metabolism. Studies suggest that polyphenols can stimulate GLP-1 secretion, helping to manage insulin resistance, hyperglycemia, and type 2 diabetes (Dao et al., 2011). Recent studies have demonstrated that botanical extracts emulate the actions of gut insulinotropic hormones. For example, PCs inhibit DPP-4 (the catabolic enzyme of GLP-1) expression, and tetrameric PCs cinnamon tannin A2 enhances GLP-1 secretion within 1 hour of ingestion (Noemi et al., 2012; Eun‐Jung et al., 2011). Meanwhile, researchers noted that oligomers and polymers of PCs enhance GLP-1 activity and insulin secretion independently of glucose loading. This finding indicates that PCs may target L cells in the intestines as a crucial regulator of blood glucose, though the exact mechanism is yet to be elucidated and warrants further investigation (Yoko et al., 2019).

In summary, PCs regulate the function of glucose-targeting organs, modulate glucose metabolism and synthesis, and maintain glucose homeostasis through multiple pathways. However, the above studies have not clarified the differences in efficacy between PCs structures and total extracts, nor the specific targets of individual metabolites. Therefore, future research should focus on these aspects to provide more targeted options for clinical applications.

### 4.4 Regulation of pancreatic beta cell function

Insulin secretion by pancreatic β-cells is crucial for preserving glucose metabolic homeostasis. Research indicates that PCs modulate islet fibrosis, enhance pancreatic β-cell function and morphology, and promote insulin release in Wistar rats with high-fat-diet-induced diabetes (Anna et al., 2012). The amount of insulin secreted by β-cells hinges on the number of insulin-producing cells. Under insulin-resistant conditions, a direct relationship emerges between β-cell count and insulin demand. Often, a persistent imbalance between β-cell proliferation and cell death sets the stage for type 2 diabetes (Laura et al., 2007). The influence of PCs on apoptosis varies among different cell types. Research has shown that PCs display chemopreventive properties in cancer cells by enhancing apoptosis and suppressing proliferation (Giovanna et al., 2024). Similarly, PCs demonstrate parallel effects on pancreatic β-cell lines. Lídia et al. (2013) established an *in vitro* model of insulin resistance and diabetes using the pancreatic INS-1E β-cell line. This approach involved subjecting INS-1E β-cells to high glucose and fatty acid conditions to induce dysfunction. The researchers investigated how elevated glucose, fatty acids, and PCs affect glucose- and palmitate-induced apoptosis in INS-1E cells. They found that PCs amplified glucose-induced apoptosis yet did not alter palmitate-induced β-cell apoptosis. This discrepancy likely arises because these two triggers engage different mechanisms underlying INS-1E cell dysfunction. Research indicates that elevated glucose levels diminish glucokinase (Gck) protein expression in pancreatic islet cells, limiting its interaction with voltage-dependent anion channels (VDAC) in the mitochondrial outer membrane. Conversely, palmitic acid triggers apoptosis via LC-CoA or other metabolites, in conjunction with endoplasmic reticulum stress and mitochondrial dysfunction. The results suggest that PCs augment high glucose-induced apoptosis in pancreatic β-cells by increasing glucose uptake under high glucose conditions, potentially via a mechanism that downregulates Gck and promotes Bax protein translocation and oligomerization at the mitochondrial membrane. Furthermore, the study showed that PCs curtail cell proliferation induced by glucose, insulin, and palmitate, although the precise mechanism remains unclear. Meanwhile, epigallocatechin gallate (EGCG), used alone or together with GLP-1 agonist exendin-4, was shown to enlarge islet area and number, expand β-cell area, and boost pancreatic insulin content in diabetic and obese mouse models, thus demonstrating favorable therapeutic outcomes (Nupur et al., 2018).

The onset of diabetes and its associated complications are tightly linked to oxidative stress and inflammation. Elevated amounts of oxidative stressors and inflammatory cytokines are closely associated with pancreatic harm in diabetic laboratory rats. Therefore, reducing oxidative stress and cytokines could represent one pathway through which PCs safeguard pancreatic islet cells. Nrf2 is pivotal in the regulation of intracellular antioxidant defense mechanisms. Mahmoud MF and his team (Mona et al., 2021) noted significant elevations in Nrf2 and HO-1 levels within the pancreatic tissues of rats suffering from STZ-induced diabetes. Studies have demonstrated that catechin-rich PCs improve the functionality of isolated pancreatic β-cells from Wistar rats and primary rat islet cells. This improvement is realized by enhancing insulin secretion through a non-oxidative stress pathway involving the activation of MAPK/PI3K to phosphorylate Nrf2, facilitating its nuclear translocation, binding to the ARE promoter, and ultimately boosting antioxidant gene expression (Thomas et al., 2017). Ji‐Hyun et al. (2009) showed that cytokines intensify inflammatory damage in isolated pancreatic islet cells, leading to disrupted insulin secretion. This result is attained by increasing nitric oxide (NO) production, enhancing NF-κB DNA binding, boosting inducible nitric oxide synthase (iNOS) expression, and activating the nuclear factor kappa-light-chain-enhancer of activated B cells (NFκB) inflammatory pathway, leading to DNA damage and elevated levels of FAS and IL-1β. Wild PCs has successfully inhibited the phosphorylation of MAPKs and PI3K/Akt, thereby preventing lipopolysaccharide (LPS)-induced NFκB nuclear translocation. As a result, this leads to diminished production of NO, prostaglandin E2 (PGE2), and ROS, along with decreased levels of inducible iNOS, cyclooxygenase-2 (COX-2), and cytokines (Min-Ji et al., 2013).

The NOD-like receptor family pyrin domain containing 3 (NLRP3) comprises a large molecular weight protein assembly, incorporating NLRP3, ASC, and caspase-1, which forms an inflammatory vesicle. This complex is responsible for releasing proinflammatory cytokines Interleukin-1β (IL-1β) and Interleukin-18 (IL-18), fulfilling a critical function in regulating insulin resistance (Bo-Zong et al., 2015). Research has shown that silencing NLRP3 inhibits obesity-induced activation of inflammatory vesicles in adipose and hepatic tissues, thereby improving insulin sensitivity—a significant finding in this area (Bolormaa et al., 2011). Initiation of the NF-κB pathway is crucial for regulating the NLRP3 inflammasome, a vital element of the innate immune system. NF-κB is acknowledged as the primary mediator responsible for transcribing NLRP3 and generating precursors of IL-1β and IL-18 (Yuan et al., 2016). Yao et al. (2022) demonstrated that PCs treatment effectively reduces NF-κBp65 nuclear translocation and NLRP3 inflammasome activation, thereby inhibiting excessive NF-κB pathway activation due to high in sugar and fat. The intervention led to a marked decrease in the concentrations of inflammatory markers like IL-6, tumor necrosis factor-α, (TNF-α), IL-17, and CRP, and notably improved insulin sensitivity in gestational diabetic mice.

In summary, PCs can regulate pancreatic β-cell function and influence insulin secretion through apoptosis, oxidative stress, and inflammatory pathways. However, the retrieved literature mainly focuses on pancreatic cell lines, with limited studies on other cell lines. Future research could establish diverse cell models to investigate whether PCs exhibit similar effects on other cell lines (Figure 5).

## 5 Protective effects of PCs on diabetes mellitus and complications

### 5.1 PCs and type 1 diabetes mellitus

Type 1 diabetes originates as an autoimmune disease involving the targeted destruction of pancreatic islet β-cells due to specific immune reactions. The β-cell primarily serves as the organ responsible for insulin secretion. Besides producing insulin, β-cells sense glucose levels and release insulin to maintain these levels within the normal physiological range (Jeffrey et al., 2010). Consequently, β-cells function not only as insulin producers but also as regulators—or “thermostats”—of glucose levels. Research indicates that key factors behind pancreatic islet β-cell dysfunction and structural damage include the presence of infiltrating CD4^+^ and CD8^+^ T cells, along with macrophages (Alan et al., 1991). When β-cells are depleted, individuals with type 1 diabetes lose glycemic control, resulting in acute conditions like ketoacidosis and severe hypoglycemia, as well as chronic complications such as heart disease, blindness, and kidney failure (Maahs and Rewers, 2006). Hence, targeting macrophages may represent a promising strategy for managing type 1 diabetes.

**FIGURE 6:**
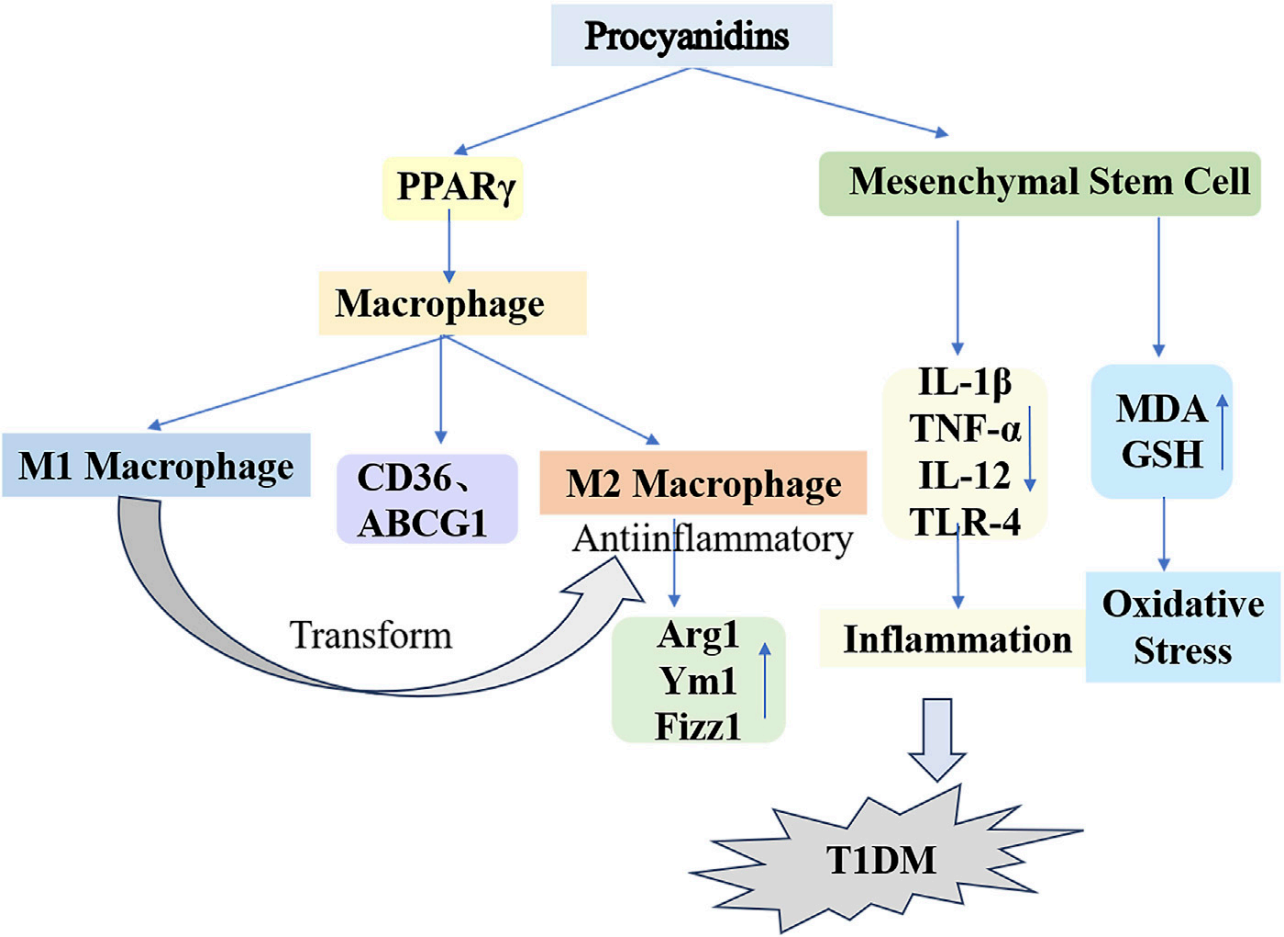
Mechanisms of Procyanidins in Counteracting Type 1 Diabetes Mellitus. This figure illustrates how PCs reduce T1DM progression by modulating macrophage polarization and mesenchymal stem cell activity. PCs promote the shift from pro-inflammatory M1 macrophages to anti-inflammatory M2 macrophages, marked by increased Arg1, Ym1, and Fizz1 expression. Additionally, PCs reduce oxidative stress and inflammation by regulating cytokines (e.g., IL-1β, TNF-α, IL-12) and enhancing antioxidant defenses (e.g., GSH, MDA).

Macrophages, key components of the innate immune system, maintain immune homeostasis and contribute to overall health. This regulatory function occurs via macrophage polarization in response to both internal and external environmental or pathological stimuli (Guillermo Arango and Albert, 2014). Ying et al. (2019) applied different concentrations of PCs to macrophages from db/db diabetic mice. Their findings indicated that PCs reduced M1 macrophage counts while elevating M2 macrophage markers such as Arginase 1, Ym1, and Fizz1. The proposed mechanism involves PCs activating peroxisome proliferator-activated receptor γ (PPARγ) in macrophages, upregulating PPARγ target genes (CD36 and ABCG1) and driving the transition from M1 to M2 phenotypes. Recently, bone marrow mesenchymal stem cells (MSCs) have been recognized as a promising treatment alternative for diabetes. However, challenges such as oxidative stress and inflammation significantly limit the efficacy of bone marrow MSC transplantation therapy. The antioxidant and anti-inflammatory properties of PCs were validated through *ex vivo* experiments (Shuang et al., 2017; Min-Ji et al., 2013). Alyaa et al. (2022) administered a daily 300 mg/kg dose of PCs combined with bone marrow MSC transplantation to STZ-induced type 1 diabetic rats over a 30-day period, resulting in successful blood glucose management and enhanced insulin secretion. Furthermore, PCs mitigated oxidative stress and lowered the concentrations of inflammatory markers such as IL-1β, TNF-α, IL-12, and TLR-4, which were elevated by MSCs, while upregulating critical antioxidant enzymes, notably glutathione (GSH). This finding demonstrates that PCs provide substantial protection against oxidative stress and inflammatory responses triggered by diabetes and MSC differentiation (Figure 6).

In conclusion, the pathogenesis of type 1 diabetes involves the immune-mediated destruction of β-cells and abnormal activation of immune cells, with macrophage polarization playing a critical role in immune regulation, making it a promising therapeutic target. PCs, with their antioxidant and anti-inflammatory properties, can amplify the therapeutic effects of bone marrow mesenchymal stem cell combination therapy, alleviating oxidative stress and inflammatory responses. Although existing studies support its therapeutic potential, further investigation is necessary to clarify its mechanisms, optimize bioavailability, and identify specific targets, while large-scale clinical trials remain imperative to validate its efficacy.

### 5.2 PCs and type 2 diabetes mellitus

Type 2 diabetes mellitus (T2DM) is recognized as a metabolic syndrome, primarily marked by insufficient insulin caused by pancreatic β-cell dysfunction and insulin resistance in target organs (NCD Risk Factor Collaboration NCD-RisC, 2016; Sudesna et al., 2017). Multiple genes govern β-cell differentiation and function—for instance, GLP-1, which enhances insulin secretion while reducing glucagon secretion; PAX4, essential for islet development; and PDX1, vital for β-cell differentiation, growth, and the maintenance of mature function. Furthermore, elements including NF-κB, Toll-like receptors, and bone bridging proteins significantly affect insulin resistance, all of which are regulated by epigenetic mechanisms (Karina et al., 2021). Yoko et al. (2019) administered various doses of cocoa-derived proanthocyanidin extracts to ICR mice, observing that PCs with distinct structures enhanced GLP-1 secretion and reduced postprandial hyperglycemia. The probable pathway likely involves PCs augmenting GLUT4 translocation and glucose uptake in skeletal muscle through an AMPK-dependent mechanism, subsequently enhancing GLP-1–activated insulin signaling and lowering postprandial hyperglycemia. *In vitro* studies show that a 10 µM concentration of PCs optimally attenuates bisphenol-induced islet cell damage, suppresses apoptosis, and improves insulin secretion. In bisphenol-induced T2DM mice, PCs reduced lipid peroxidation, boosted antioxidant enzymes (SOD and Gpx), upregulated mRNA levels of PDX1 and GUT2, and preserved glucose homeostasis. Furthermore, combined catechin and quercetin therapy demonstrated a stronger impact on elevating key enzymatic activities in hepatic glucose metabolism in diabetic rats relative to monotherapy (Byoung Rai and Pyoung Sim, 2022). Additionally, a double-blind clinical trial reported that continuous intake of a PCs-rich beverage, largely composed of catechin, stimulated insulin secretion and enhanced blood glucose control in individuals with type 2 diabetes (Tomonori et al., 2009). Collectively, these findings prevented BPA-induced islet cell apoptosis, hyperglycemia, and diabetic onset (Ahangarpour et al., 2016).

Insulin resistance is a major contributor to the development of type 2 diabetes. Furthermore, mitochondrial dysfunction plays a pivotal role in the progression of insulin resistance. Essential for maintaining normal mitochondrial function, mitochondrial biosynthesis is regulated by key factors such as Peroxisome receptor gamma coactivator 1-alpha (PGC-1α) and silent information regulator factor 2-related enzyme1 (SirT1). PGC-1α acts as a coactivator for transcription factors that regulate mitochondrial genes, including nuclear respiratory factor 1 (NRF1) and mitochondrial transcription factor A (TFAM). Conversely, SirT1-mediated deacetylation activates PGC-1α (Helena et al., 2009; Bor Luen, 2016). In a dexamethasone-induced insulin resistance model using 3T3-L1 adipocytes, flavan-3-ols from PCs enhanced the expression of genes involved in mitochondrial biosynthesis (PGC-1α, SirT1, NRF1) and fusion proteins (Mfn1 and Mfn2). Furthermore, PCs reduced the expression of the fission protein Drp1, thereby improving mitochondrial defects in 3T3-L1 adipocytes by enhancing mitochondrial biosynthesis, dynamics, membrane potential, and antioxidant capacities, ultimately mitigating insulin resistance (Fangfang et al., 2020).

**FIGURE 7:**
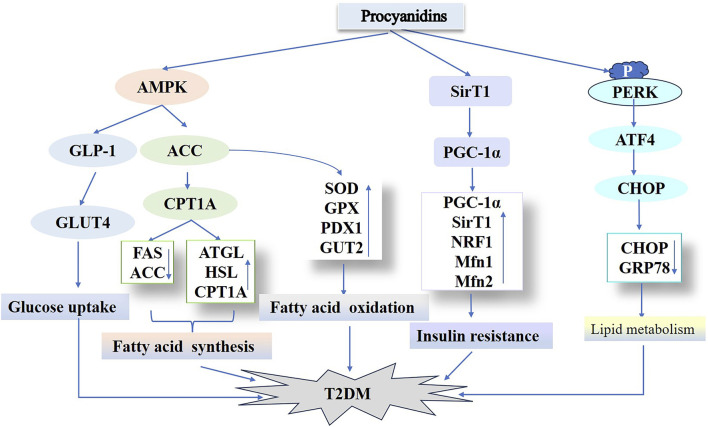
Procyanidins’ Role in Regulating Type 2 Diabetes Mellitus Pathways. This figure highlights how PCs improve T2DM by enhancing glucose uptake through AMPK activation and GLUT4 translocation, while modulating lipid metabolism via ACC, ATGL, and CPT1A. PCs also improve insulin sensitivity and reduce insulin resistance by regulating SIRT1, PGC-1α, and NRF1. Additionally, PCs mitigate endoplasmic reticulum stress and associated T2DM progression through pathways involving PERK and CHOP.

Hyperlipidemia substantially contributes to type 2 diabetes susceptibility. This condition arises from disrupted hepatic lipid metabolism, characterized by elevated triglyceride synthesis, increased cholesterol production, and reduced fatty acid oxidation. These processes drive excessive hepatic fat accumulation, culminating in steatosis (Lewis et al., 2002; David et al., 2007). Thus, optimizing lipid metabolism represents a viable strategy for both preventing and managing type 2 diabetes. In their research, Fangfang et al. (2020) used PCs to treat a T2DM mouse model induced by a high-fat diet (HFD) and STZ. PCs treatment significantly decreased hyperglycemia and hyperinsulinemia, as well as serum triglycerides, total cholesterol, LDL cholesterol, and AST-associated lipokines in T2DM mice compared to controls. PCs treatment enhanced diabetic hyperlipidemia and liver function in mice. Additionally, PCs suppressed adipogenic proteins (FAS, ACC) while upregulating lipolytic proteins (ATGL, HSL, CPT1A). The results indicate that PCs may suppress lipogenesis while enhancing lipid hydrolysis and β-oxidation processes for fatty acids. This effect likely occurs via AMPK/ACC/CPT1A pathway activation, enhancing hepatic lipid metabolism in HFD/STZ-induced diabetic mice. The degree of polymerization in PCs correlates with their biological potency. Zhao et al. (2024) administered equivalent doses of PCs exhibiting varying polymerization levels to HFD/STZ-induced diabetic mice. These PCs significantly lowered fasting blood glucose, improved glucose/insulin tolerance, and optimized lipid profiles (total cholesterol, triglycerides, LDL cholesterol), while reducing oil-red staining in hepatic and serum tissues. They significantly downregulated CHOP and GRP78 expression in diabetic mouse livers, implying a link between hepatic lipid dysregulation and endoplasmic reticulum stress. Furthermore, *in vitro* experiments showed that administering PCs of different polymerization degrees to palmitic acid–induced HepG2 cells markedly diminished intracellular GRP78 expression. Additionally, this approach activated the protein kinase R-like endoplasmic reticulum kinase (PERK)/active transcription factor 4 (ATF4) signaling pathway, crucial for endoplasmic reticulum stress regulation, and partially mitigated hepatic lipid accumulation. These findings align with earlier investigations in high glucose–induced C57BL/6 mice treated with PB2, indicating that PCs regulate lipid metabolism in diabetes by targeting endoplasmic reticulum sensors such as PERK, IRE1, and ATF6 (Liu Z. et al., 2023; Nie et al., 2020). GSPE with varying polymerization degrees exhibit protective activity on blood glucose, lipid levels, and hepatic oxidative stress in diabetic rats (Zhaoxia et al., 2014). Notably, oligomers outperformed polymers, implying that the oligomeric form of GSPE might provide superior benefits over other forms. In conclusion, PCs and their derivatives, spanning different polymerization degrees, may influence the initiation and progression of T2DM via multiple pathways. Hence, these plant metabolites show promise as active agents for type 2 diabetes management (Figure 7).

In summary, PCs participate in T2DM pathophysiology via multiple mechanisms, such as enhancing insulin secretion, safeguarding pancreatic β-cells, optimizing mitochondrial function and insulin resistance, and regulating hepatic lipid metabolism. Their polyphenolic metabolites display significant potential in regulating endoplasmic reticulum stress and lipid metabolism. As natural plant metabolites, PCs hold considerable promise as a therapeutic option for T2DM, but their specific mechanisms and clinical applications demand further research.

### 5.3 PCs and gestational diabetes mellitus

Gestational diabetes mellitus (GDM) emerges exclusively during pregnancy or is diagnosed when abnormal glucose tolerance is first identified (Sweeting et al., 2022). The pathogenesis of GDM is intricate, primarily involving impaired insulin secretion and reduced functionality. Insulin resistance gradually intensifies during pregnancy, ultimately leading to hyperglycemia (Jasmine et al., 2018; Kimber-Trojnar et al., 2018). Glucose, which traverses the placenta, serves as the primary energy source for the developing fetus (Ruszała et al., 2022). Furthermore, the overactivation of adipose tissue, which produces monocytes and macrophages that secrete inflammatory factors, contributes to the pathogenesis of GDM by initiating inflammatory responses. Growing evidence indicates that abnormal inflammatory responses are major contributors to the development of insulin resistance (Skórzyńska-Dziduszko et al., 2016; Akash et al., 2018; Gao et al., 2016). In a study utilizing a GDM mouse model induced by a high-fat diet and sugar, PCs (27.8 mg/kg/d) were administered from 4 weeks before pregnancy until delivery to evaluate their impact on insulin resistance, including during the postnatal period and in offspring. PCs effectively managed weight gain during pregnancy, decreased serum fasting blood glucose (FBG), fasting insulin, oral glucose tolerance test area under the curve, and insulin tolerance test area under the curve levels, and enhanced the Homeostatic Model Assessment (HOMA) insulin sensitivity index. These interventions positively affected fasting glucose and insulin levels. Additionally, PCs improved glucose metabolism and reduced concentrations of inflammatory mediators such as IL-6, TNF-α, IL-17, and CRP, thereby diminishing inflammation and hepatic inflammatory infiltration in mice 4 weeks postnatally. However, these treatments did not significantly impact the offspring. In a related observation, PCs were used in a GDM mouse model with gut flora defects induced by a combination of broad-spectrum antibiotics and a high-fat, high-sugar diet. This treatment enhanced insulin resistance by inhibiting the NF-κB/NLRP3 activation pathway, thereby altering the levels of gut flora metabolites 4-hydroxyphenylacetic acid and 3- (4-hydroxyphenyl) propionic acid (Yao et al., 2022). In a double-blind, randomized, controlled trial on gestational diabetes (Zhang et al., 2017), it was observed that mothers in the epigallocatechin 3-gallate group showed significant improvements in diabetes parameters, with fewer cases of neonatal complications compared to the placebo group.

In conclusion, PCs exert significant protective effects on the pathogenesis of GDM by enhancing insulin sensitivity, lowering levels of inflammatory factors, and alleviating liver inflammation, while effectively regulating glucose metabolism during pregnancy and *postpartum* stages. Despite limited effects on offspring mice, PCs continue to show potential as a treatment for GDM, but their long-term safety and mechanisms require further research and verification.

### 5.4 Effect of PCs on diabetic complications

#### 5.4.1 PCs and diabetic nephropathy

Diabetic nephropathy (DN) is a primary microvascular complication of diabetes mellitus, characterized by proteinuria, glomerular enlargement, reduced tubular filtration, renal fibrosis, and renal dysfunction, ultimately leading to end-stage renal disease (Samsu, 2021). The progression of diabetic nephropathy is multifaceted, influenced by factors such as renal hemodynamic abnormalities, dyslipidemia, oxidative stress, and hormone synthesis, including Angiotensin II (Ang II). Additionally, its progression is influenced by inflammation, the renin-angiotensin system (RAAS), advanced glycation end-product (AGE) formation, and signaling molecules, including transforming growth factor-β1 (TGF-β1), connective tissue growth factor (CTGF), PKC, MAPK, and ROS. Collectively, these factors accelerate the progression of diabetic nephropathy, making it a leading cause of end-stage renal disease (Javier et al., 2020; Su Woong and Jae‐Young, 2021; Majid, 2013; Wei‐Jian et al., 2015; Yashpal et al., 2011; Mandeep Kumar and Umesh Kumar, 2013). In a rat model induced by STZ, a 12-week regimen of PCs treatment resulted in improvements in proteinuria and podocyte injury. Its probable mechanism includes activation of renal AGE-RAGE signaling pathways, decreasing levels of monocyte chemotactic protein-1 (MCP-1) and PKC-A, which consequently reduces the production of slit diaphragm proteins such as nephrin and podocin, thereby mitigating the impact of AGE-mediated DN pathogenesis (Muthenna et al., 2014). Oral administration of PCs significantly enhanced kidney function and reduced pathological alterations in db/db mice. Proteomic evaluation revealed that following PCs administration, 53 proteins were downregulated and 60 upregulated, with milk fat globule-epidermal growth factor 8, (MFG-E8) as the most notably upregulated protein in diabetic kidneys. Inhibition of MFG-E8 via shRNA transfection decreased phosphorylation levels of ERK1/2, Akt, and GSK-3β in the kidneys of db/db mice, improving renal histopathology. Conversely, overexpression of MFG-E8 had opposite effects. PCs treatment reduced MFG-E8, phospho-ERK1/2, phospho-Akt, and phospho-GSK-3β levels in db/db mouse kidneys, indicating MFG-E8’s influence on the progression of diabetic nephropathy. PCs appear to mitigate diabetic nephropathy by reducing MFG-E8 levels and modulating the ERK1/2, Akt, and GSK-3β pathways (Zhang et al., 2013).

**FIGURE 8:**
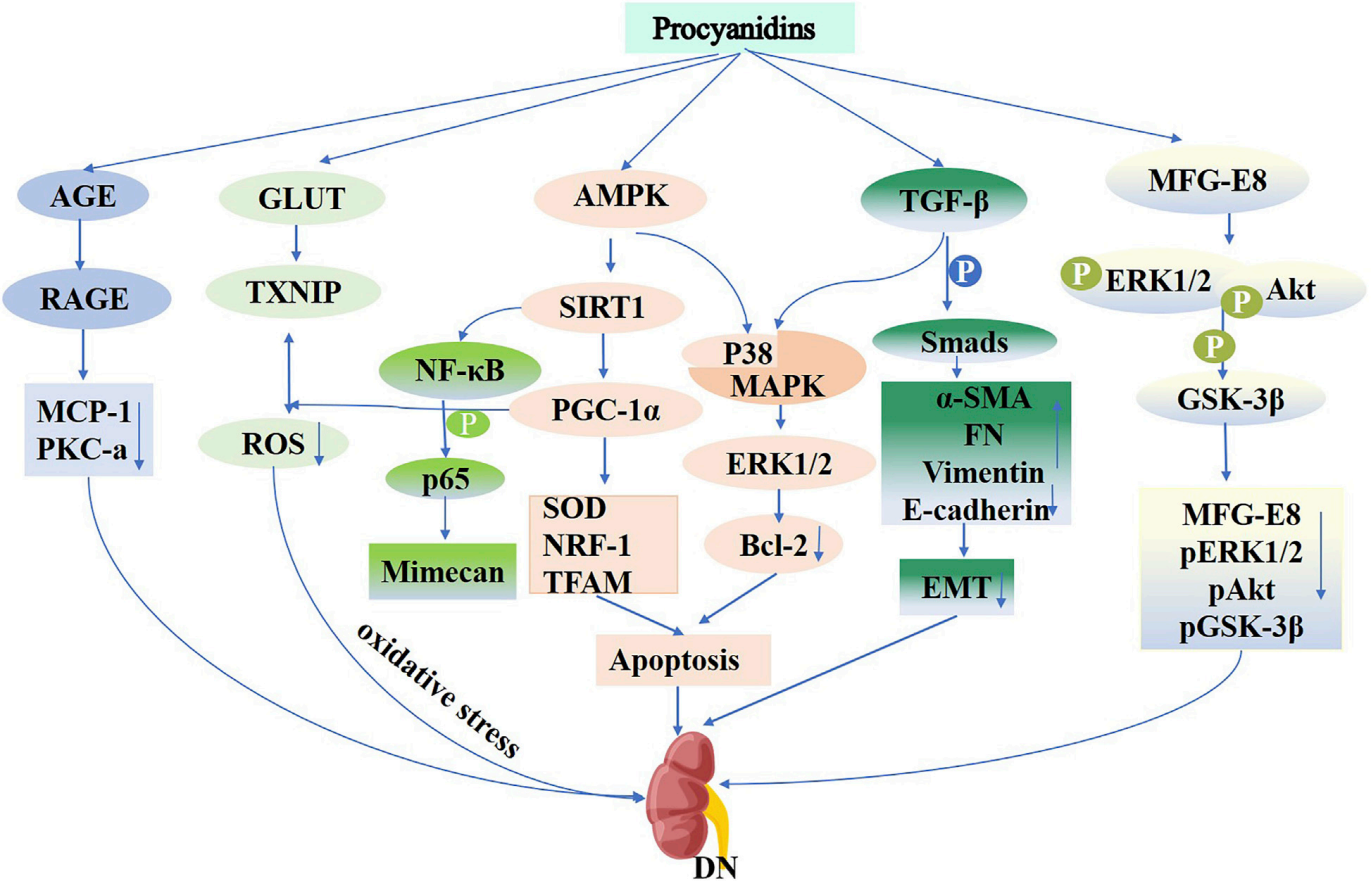
Mechanisms of Procyanidins in Diabetic Nephropathy. This figure highlights how PCs combat DN by reducing oxidative stress through AMPK activation and GLUT-mediated glucose uptake. PCs inhibit inflammation via the NF-κB and PKC-α pathways and attenuate renal fibrosis by modulating TGF-β/Smad signaling. Additionally, PCs prevent epithelial-to-mesenchymal transition (EMT), preserving renal cell integrity and reducing apoptosis, thereby mitigating disease progression.

Diabetic glomerulosclerosis is a defining feature of diabetic nephropathy. A key process in glomerulosclerosis and tubular epithelial fibrosis involves the epithelial-to-mesenchymal transition (EMT) induced by high glucose levels (Xu et al., 2017). PCs mitigate high glucose-triggered EMT within HK-2 cells by activating the TGF-β/Smads and P38/MAPK pathways, upregulating α-SMA, FN, and Waveform proteins, and downregulating E-cadherin levels (Li et al., 2015). Oxidative stress plays a crucial role in the progression of diabetic kidney injury. Research shows that PCs protect cells from the systemic high glucose impacts on mitochondria, preventing cellular dysfunction and cell death by activating the AMPK-SIRT1-PGC-1α pathway. This compound reduces ROS levels, enhances SOD antioxidant activity, and increases the expression of NRF-1 and TFAM genes (Cai et al., 2016). In diabetic nephropathy models, PCs attenuated renal cell apoptosis by enhancing p38 MAPK and ERK1/2 activities and reducing Bcl-2 expression. GSPE also reduced thioredoxin-interactingprotein expression, facilitating intracellular ROS catabolism via the sulfhydryl antioxidant pathway to mitigate oxidative stress in diabetic kidneys (Wei et al., 2018). Additionally, PCs alleviated DN pathology, such as glomerular basement membrane thickening and central dilatation, by modulating mimecan protein expression through the NF-κB p65 pathway (Zhou et al., 2016) (Figure 8). The combined treatment of perindopril and catechin significantly improved mesangial matrix and podocyte function in diabetic rats, effectively preventing glomerular injury, with greater efficacy compared to monotherapy (Salime Pelin et al., 2014). A clinical study demonstrated (Tung-Sheng et al., 2011) that a 1:1 mixture of epigallocatechin-3-gallate and Amla (Emblica officinalis) significantly enhanced antioxidant defenses in patients with diabetic nephropathy, synergistically improving clinical outcomes.

PCs and their metabolites exhibit significant therapeutic potential in the pathogenesis of diabetic nephropathy through multiple mechanisms, including antioxidant and anti-fibrotic effects, as well as the regulation of key signaling pathways. They are particularly effective in reducing proteinuria, protecting mitochondrial function, and inhibiting inflammatory responses. Despite these promising findings, current research is largely confined to animal models and *in vitro* studies, with limited clinical investigations available. Therefore, further large-scale clinical trials are essential to confirm the safety and efficacy of PCs in managing diabetic nephropathy, thereby establishing a foundation for their clinical application.

#### 5.4.2 PCs and diabetic retinopathy

Diabetic retinopathy (DR) is a significant microvascular complication of diabetes mellitus and a leading cause of vision loss worldwide (Tan and Wong, 2022; Liu and Wu, 2021; Joanne et al., 2012). DR stems from a complex pathology involving oxidative stress, inflammatory lesions, and the polyol pathway. Activation of hexosamine flux and advanced glycation end product (AGE) formation, driven by PKC isoform activation and related pathways, contributes to vascular dysfunction and tissue injury in the retina (Yau et al., 2012; Al-Shabrawey et al., 2015; Lu et al., 2018). Early and timely intervention is crucial to prevent retinal damage and vision impairment in individuals with diabetes.

**FIGURE 9:**
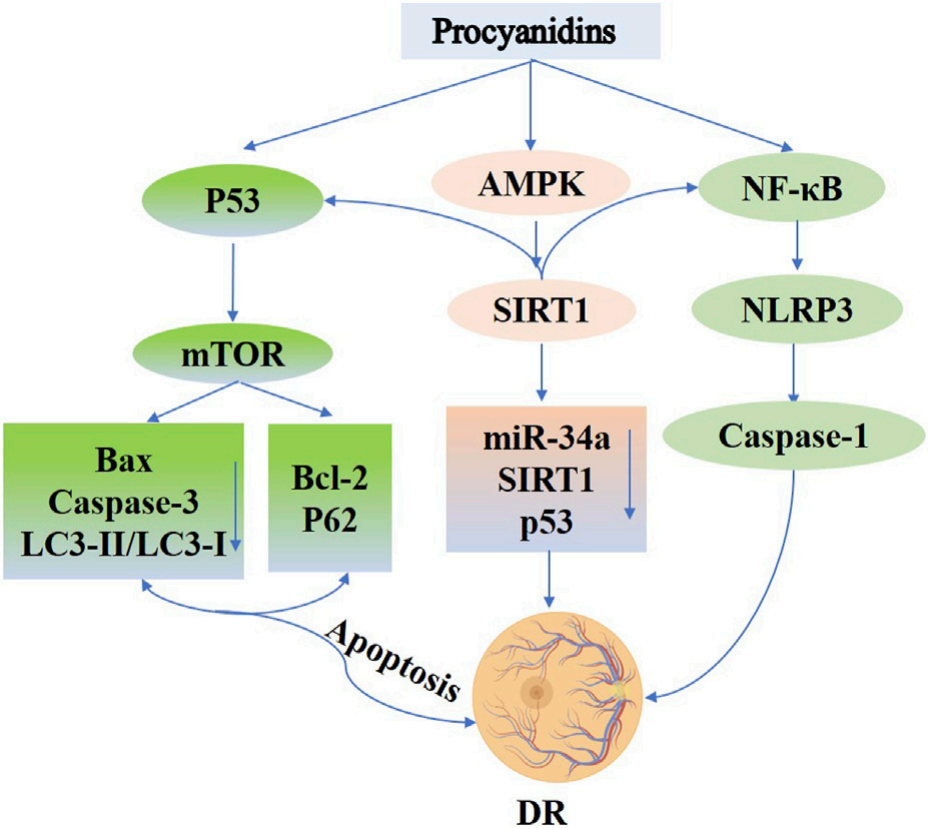
Procyanidins’ Role in Diabetic Retinopathy. This figure highlights how PCs alleviate DR by modulating key signaling pathways. PCs activate AMPK and SIRT1 to reduce oxidative stress and regulate apoptosis via p53, Bax, and Caspase-3. PCs also promote autophagy through mTOR signaling and inhibit inflammation by suppressing NF-κB and NLRP3 activation. Additionally, PCs regulate miR-34a and SIRT1 to protect retinal cells, reducing apoptosis and inflammation, thereby mitigating the progression of diabetic retinopathy.

Recent studies indicate that PCs exert protective effects on retinal cells by modulating autophagy, reducing oxidative stress, and attenuating inflammation (Rodríguez-Carrizalez et al., 2014; John et al., 2020). In high-glucose–challenged retinal pigment epithelial cells, PCs activate the p53/mTOR autophagy pathway, lowering apoptosis-related factors (e.g., Bax, Caspase-3, LC3-II/LC3-I, and phosphorylated p53) and elevating the levels of survival proteins (Bcl-2, p62, and phosphorylated mTOR) (Li et al., 2022). Additionally, the application of PCs to rat retinal capillary endothelial cells (TR-iBRB2) decreases oxidative stress and inflammasome activation, suggesting involvement of the oxidase/NF-κB/NLRP3 axis in mediating their cytoprotective effects (Zou et al., 2023). Further research reveals that PCs, alone or in combination with other phytoalexins, can activate the AMPK/SIRT1/NF-κB pathway and inhibit the miR-34a/SIRT1/p53 axis, thereby mitigating hyperglycemia-induced inflammation, oxidative stress, and apoptosis in retinal cells (Liu et al., 2021) (Figure 9).

Moreover, mounting evidence highlights the anti-glycation and anti-dicarbonyl properties of PCs, which inhibit the formation of AGEs by scavenging reactive carbonyl species (e.g., methylglyoxal) (Qian et al., 2013). By reducing the burden of these glycation intermediates, PCs help maintain retinal integrity and limit the progression of DR.

In conclusion, PCs safeguard retinal cells and mitigate diabetic retinopathy through various molecular mechanisms, including autophagy activation, oxidative stress reduction, anti-glycation activity, and anti-inflammatory modulation. These pleiotropic actions underscore the potential of PCs as promising therapeutics for DR and warrant further investigation in both preclinical and clinical settings.

#### 5.4.3 PCs and diabetic neuropathy

Diabetic neuropathy (DN), commonly presenting as a distal symmetrical polyneuropathy, is characterized by sensory deficits and pain in the extremities (Eva et al., 2019). Hyperglycemia-driven pathways accelerate DN progression, with AGEs and reactive dicarbonyl compounds exacerbating nerve injury through chronic inflammation and neuronal apoptosis (Stella et al., 2021; Ryuichi et al., 2001).

**FIGURE 10:**
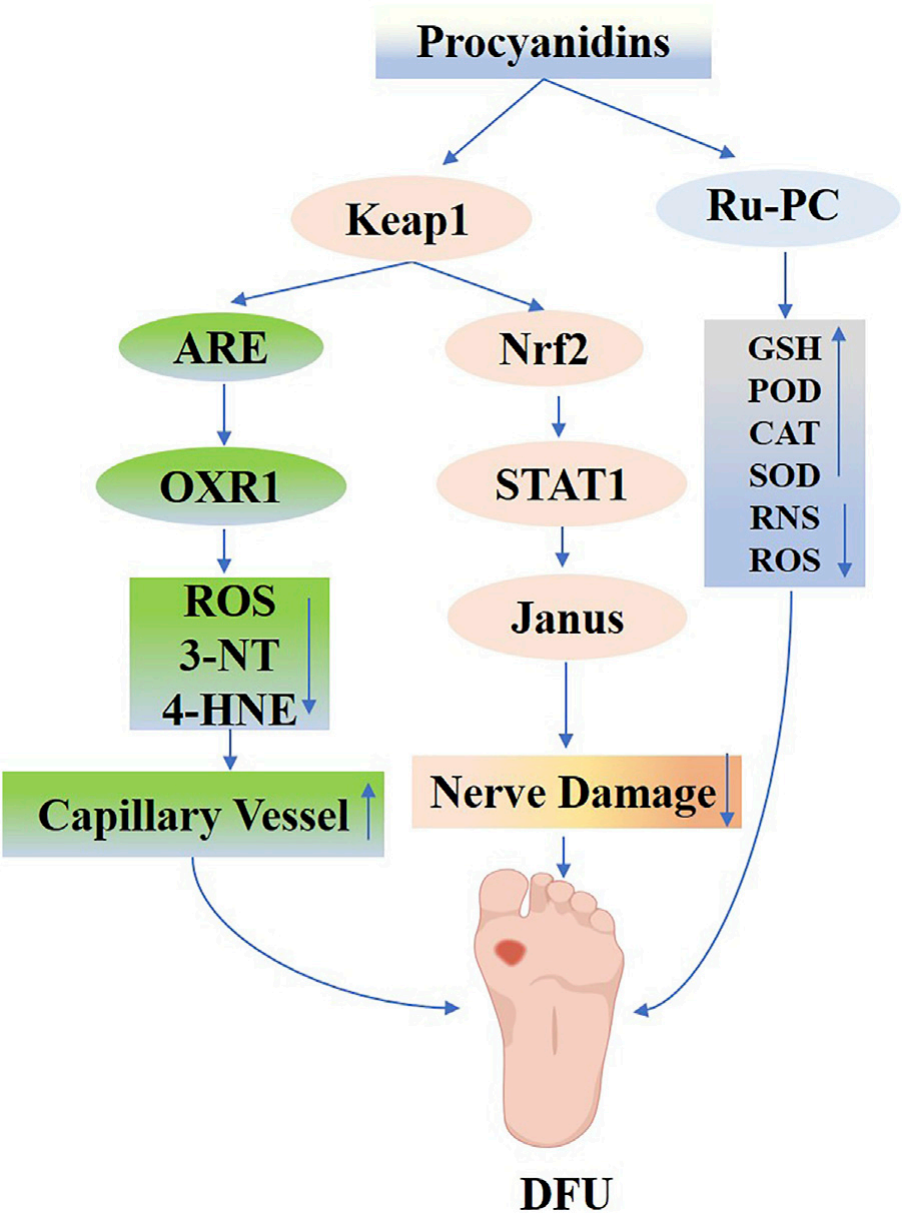
Mechanisms of Procyanidins in Diabetic Neuropathy and Diabetic Foot Ulcers. This figure shows how PCs alleviate DN and DFU by activating the Keap1/Nrf2 pathway, enhancing antioxidant defenses, and reducing oxidative damage (ROS, 3-NT, 4-HNE). PCs also regulate STAT1/JAK signaling to control inflammation and promote nerve repair while improving capillary function. Additionally, PCs support anti-inflammatory responses by modulating GSH, SOD, and other antioxidant systems, facilitating vascular and neural recovery critical for ulcer healing.

PCs have shown promising neuroprotective effects by reducing oxidative stress, preventing the accumulation of AGEs and other toxic intermediates, and mitigating glycation-induced damage. Moreover, their anti-glycation and anti-dicarbonyl properties lessen the accumulation of AGEs and other toxic intermediates, consequently protecting peripheral nerves from glycemic damage (Xiao-Fang et al., 2008; Xiao-Fang et al., 2010). Notably, in models of type 2 diabetes, PCs confer neuroprotection by modulating NF-E2/STAT1 signaling, attenuating Janus kinase activity, and inhibiting cell apoptosis, thereby reducing cerebrovascular lesions and associated neurological deficits (Wu et al., 2015; Yin et al., 2021) (Figure 10).

In summary, PCs offer a multifaceted neuroprotective effect in DN by targeting oxidative and inflammatory pathways, preventing glycation-related damage, and reducing neuronal apoptosis. Further clinical studies are needed to validate these findings and establish optimal dosing regimens for maximizing the therapeutic efficacy of PCs in managing DN.

#### 5.4.4 PCs and diabetic foot ulcer

Diabetic foot ulcers (DFU) are severe and common complications stemming from a multifactorial interplay of sensory, motor, autonomic, and vascular abnormalities, along with altered foot biomechanics (McDermott et al., 2023). Approximately 50%–60% of diabetic ulcer patients develop infections, and about 20% of these cases, involving moderate to severe infections, require lower extremity amputation (David et al., 2023). Patients with DFU face a 5-year mortality rate of about 30%, which rises to over 70% for those undergoing major amputations. Moreover, infected diabetic ulcers significantly heighten the risk of disability and mortality among these patients (Armstrong et al., 2020). Diabetic ulcer wounds that are resistant to healing markedly increase the risk of bacterial invasion and subsequent infection. Thus, prompt treatment and intervention aimed at promoting wound healing in diabetic ulcers are crucial for reducing infections and preventing amputations in diabetic patients. The wound healing process encompasses hemostasis, inflammation, proliferation, and wound remodeling. Hyperglycemia-induced cellular dysfunction, prolonged low-grade inflammation, and tissue ischemia and hypoxia are well-documented barriers to wound healing, further decelerating the repair of refractory diabetic wounds (Matoori et al., 2021).

Research increasingly indicates that plant-derived active ingredients support the differentiation and functional capabilities of various cell types, thereby promoting wound healing (Kisseih et al., 2015). Specifically, PCs have exhibited notable efficacy. Pimentel EF and their group (Pimentel et al., 2024) employed resonance mass spectrometry to analyze seed hulls, noting that PCs enhanced fibroblast functionality and migration, and increased the levels of vascular endothelial growth factor (VEGF) and platelet-derived growth factor (PDGF). These improvements supported the healing of wounds infected with *Staphylococcus aureus*. Subsequent experiments showed that PCs significantly enhanced the angiogenic, survival, and migration capabilities of human umbilical cord blood endothelial progenitor cells under high-glucose conditions, thus reducing glucose-induced damage. This protective action likely involves regulating antioxidant gene expression via Nrf2 activation, diminishing excessive ROS accumulation, and reducing oxidative damage markers (e.g., 3-NT and 4-HNE), as evidenced by J Fan et al.‘s study on STZ-induced diabetic mouse wounds treated with PCs (Fan et al., 2021). They found that PCs not only reduce oxidative stress in damaged skin via Nrf2 activation but also stimulate capillary formation and facilitate endothelial progenitor cell migration to the wound site, thereby accelerating wound healing and promoting neoangiogenesis.

The integration of nanomaterials and hydrogels with plant-derived active ingredients for wound healing has recently attracted significant interest among researchers. PCs, polyphenolic compounds derived from plants, exhibit antioxidant, antibacterial, and anti-inflammatory properties. When combined with ruthenium-liganded nano-enzymes, PCs are converted into potent antimicrobial agents that could potentially replace antibiotics in treating bacterial-infected wounds. Shan et al. (2024) combined RuCl₃ with polyvinylpyrrolidone to synthesize Ru-PVP, then mixed it with PCs in a methanol solution to formulate Ru-PC nano-enzymes exhibiting four enzymatic activities. The functional pathways of Ru-PC nano-enzymes varied significantly across different environments. In acidic environments, these nanoenzymes demonstrated effective antimicrobial properties through GSH reduction and peroxidase (POD) -mimetic activity. Under neutral conditions, Ru-PC nanoenzymes break down H2O2 into O2 via catalase (CAT) activity, thus alleviating wound healing complications due to hypoxia. Concurrently, Ru-PC exhibits SOD-like activity, efficiently eliminating excessive reactive oxygen and nitrogen species in neutral environments, thereby preserving antioxidant balance. Additionally, Ru-PC modulates the transition from M1 to M2 macrophage polarization, aiding in the management of inflammatory wound repair challenges. In conclusion, Ru-PC nanoenzymes expedite the healing of infected wounds by stimulating cell proliferation, eradicating bacteria, reducing inflammation, neutralizing free radicals, supplying oxygen, and promoting blood vessel growth. Liu K. et al. (2023) created a versatile PBOF hydrogel dressing composed of poly (vinyl alcohol) (P), borax (B), oligomeric PCs (O), and ferric ions (F). This hydrogel is characterized by its adhesive, shape-adapting, stretchable, self-healing, detachable, antioxidant, photothermal antimicrobial, hemostatic, and biocompatible properties, making it exceptionally suitable for managing wounds in highly active areas. In a mouse model with *Staphylococcus* aureus-induced cervical wound infections, the PBOF hydrogel dressing showed antimicrobial activity, utilizing the photothermal properties of ferric ions and polyphenol chelates, together with the hemostatic, antimicrobial, and anti-inflammatory qualities of PCs. These features aid in wound healing by minimizing oxidative stress, curbing inflammation, and enhancing angiogenesis (Figure 10).

In conclusion, the occurrence of DFU is closely related to cell dysfunction, low-grade inflammation, and tissue hypoxia induced by hyperglycemia. PCs significantly promote wound healing by alleviating oxidative stress, promoting endothelial progenitor cell migration, improving angiogenesis, and exerting anti-inflammatory and antibacterial effects. Moreover, the combination of nanomaterials and hydrogels with plant bioactive metabolites further enhances the therapeutic effect, suggesting that PCs hold great promise for the treatment of DFU.

### 5.5 The effects of PCs on atypical complications of diabetes

#### 5.5.1 PCs and diabetic encephalopathy

Diabetic encephalopathy (DE), a diabetes-related complication marked by central nervous system damage, primarily results from cerebral microangiopathy, including conditions such as stroke, dementia, and depression (van Sloten et al., 2020). Numerous studies have confirmed that DM independently increases the risk of conditions such as ischemic stroke, transient ischemia, and vascular dementia (Antal et al., 2022). People with type 2 diabetes have a 2.5-fold increased risk of ischemic stroke and are 1.5 times more likely to experience hemorrhagic stroke and dementia than those without diabetes (Sarwar et al., 2010; Cheng et al., 2012). Potential mechanisms linking diabetes to stroke include vascular endothelial dysfunction, atherosclerosis, inflammatory responses, oxidative stress, and alterations in blood-brain barrier permeability (van Sloten et al., 2020; Chen et al., 2016). Wu et al. (2015) employed PCs using a mouse model of middle cerebral artery occlusion (MCAO) to examine its impact. They observed that PCs reduced infarct size, cerebral swelling, and neurological impairments following MCAO. This effect is attributed to PCs’ role in facilitating nuclear translocation, enhancing levels of HO-1, GSTα, NQO1, and ZO-1 proteins in the ischemic zone to maintain blood-brain barrier integrity and mitigate damage to the nervous system. Similarly, PCs activated the TLR4-p38-NF-κB-NLRP3 signaling pathway, which decreases the production of inflammatory cytokines in response to MCAO/R and OGD/R both *in vivo* and *in vitro*. Furthermore, it exhibited neuroprotective properties by lessening cerebral edema, reducing infarct size, minimizing histological damage, and decreasing microglial cell death (Yang et al., 2020).

**FIGURE 11:**
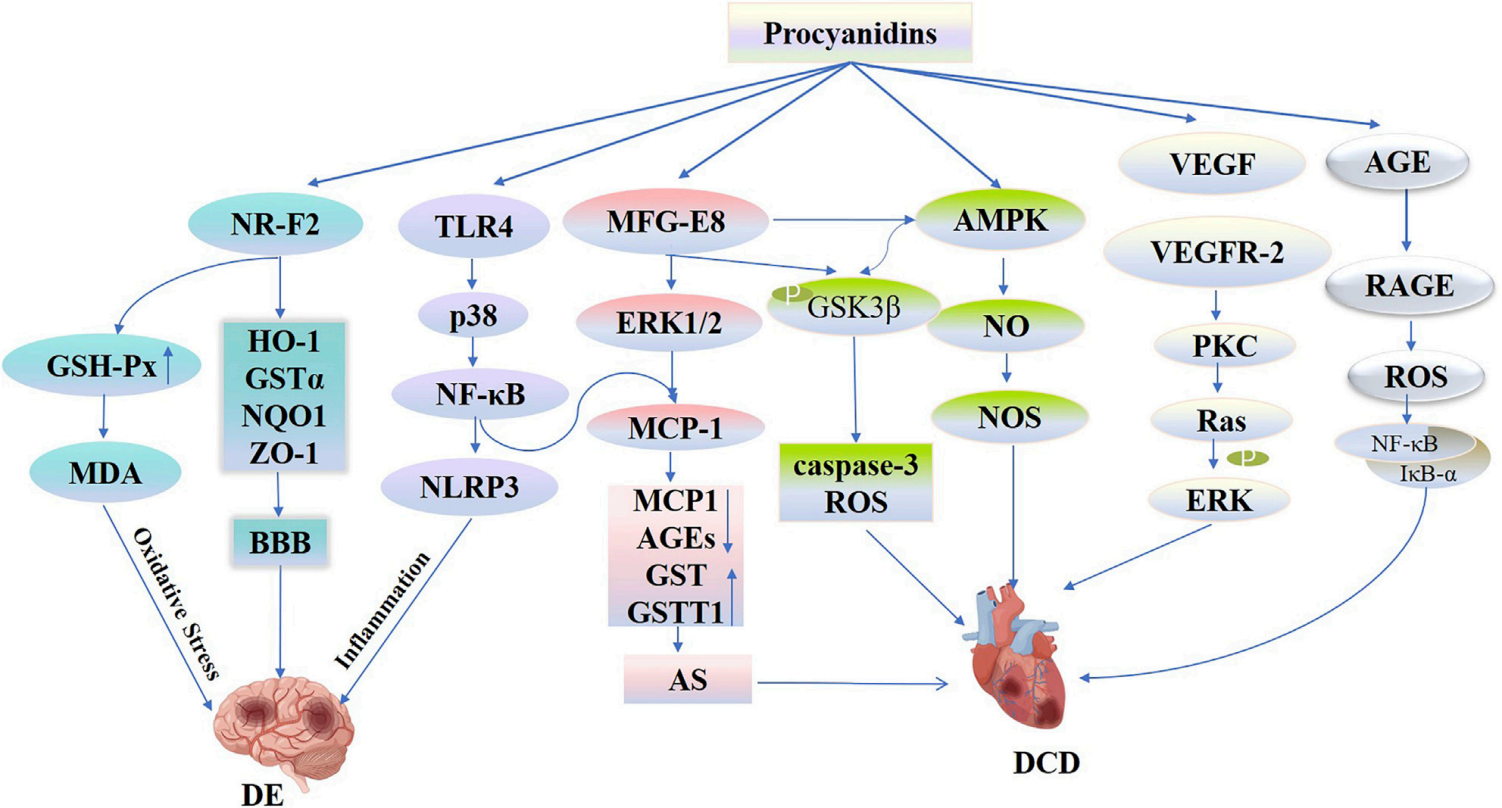
Procyanidins’ Mechanisms in Diabetic Encephalopathy and Cardiovascular Disease. This figure illustrates how PCs mitigate DE and DCD through multiple pathways. PCs activate NRF2 signaling, increasing antioxidant enzymes (e.g., HO-1, GSTα, NQO1) and strengthening the blood-brain barrier (BBB) to reduce oxidative stress and inflammation in DE. PCs also regulate the TLR4-p38-NLRP3 pathway to suppress neuroinflammation. In the cardiovascular system, PCs modulate MFG-E8-ERK1/2 and AMPK-NO-NOS pathways to reduce MCP-1 levels, oxidative stress, and apoptosis, improving vascular function and preventing atherosclerosis and DCD progression.

Oxidative stress acts as a critical factor in the onset of ischemic reperfusion damage. PCs derived from grape seeds significantly modulate oxidative stress responses. In experiments employing the MCAO/R model, PCs significantly reduced neurological impairment scores at various intervals—1, 24, 72 h, and 7 days post-MCAO. Additionally, PCs effectively reduced the volume of cerebral infarcts and curbed malondialdehyde (MDA) levels in brain tissues. Significantly, PCs lowered MDA levels and boosted glutathione peroxidase (GSH-Px) activity in brain tissues relative to the control group. This protective effect was achieved by mitigating oxidative stress and apoptosis, promoting angiogenesis, and triggering the GSH-Px antioxidant pathway, thus defending against ischemia-reperfusion brain injury (Kong et al., 2017) (Figure 11).

In conclusion, PCs demonstrate significant neuroprotective effects through their antioxidant, anti-inflammatory properties, and maintenance of blood-brain barrier function. Additionally, PCs provide effective protection against cerebral ischemia-reperfusion injury by alleviating oxidative stress, promoting angiogenesis, and activating antioxidant enzyme pathways. Both provide potential therapeutic strategies for treating brain injury caused by diabetes, but their clinical application requires further validation.

#### 5.5.2 PCs and diabetic cardiovascular disease

Diabetic cardiovascular disease (DCD) remains the primary cause of mortality among individuals with type 2 diabetes. Dysfunction of vascular endothelial cells is an initial contributor to cardiovascular disease (Strain and Paldánius, 2018). Nitric oxide is critical for regulating vasodilation. A diminished availability of nitric oxide can lead to endothelial dysfunction (Jeong et al., 2022). Elevated glucose levels activate the enzyme aldose reductase (AR), which transforms glucose to sorbitol, with NADPH serving as a coenzyme for this process. Depletion of NADPH impairs NO synthesis and lowers glutathione levels, causing an overproduction of oxygen free radicals, which compromises endothelial cell function in blood vessels (Belenichev et al., 2023). Clinical research has shown that PCs raise endothelial nitric oxide synthase (eNOS) levels, subsequently reducing systolic and diastolic blood pressure values (Odai et al., 2019; Schön et al., 2021). Simultaneously, PCs stimulate AMPK, which increases eNOS levels and nitric oxide production, thereby enhancing vascular endothelial performance (Cui et al., 2012). Research indicates that microRNAs (miRNAs) are involved in endothelial cell dysfunction and apoptosis (Silambarasan et al., 2016). PCs modulate glucose metabolism by regulating microRNA expression in the pancreas (Castell-Auví et al., 2013). Elevated blood sugar levels impair vasodilation by activating PKC in vascular cells, affecting the activities of ET-1, VEGF, NO, PDGF, ROS, and NF-κB in pericytes, leading to capillary myelopoietic thickening and increased endothelial cell proliferation (Barrett et al., 2017). Liu et al. (2016) demonstrated that PCs and their oligomers enhance cell proliferation, reducing oxidative damage in endothelial cells under high glucose conditions. This outcome is realized by enhancing VEGFR-2 expression and activating its downstream signaling pathways. Furthermore, glycated LDL, common in diabetic patients, substantially contributes to vascular endothelial cell dysfunction and apoptosis. Proteomic research reveals that extracts from grape seed PCs suppress apoptosis in vascular endothelial cells, initiated by glycated LDL, through the modulation of PIMT, a methyltransferase involved in protein repair (Li et al., 2013).

Considerable evidence indicates that the expansion of vascular endothelial and smooth muscle cells, induced by AGEs, is vital for diabetic vasculopathy (Barbosa et al., 2008). The NF-κB signaling pathway substantially affects the movement, invasion, and growth of vascular smooth muscle cells (VSMCs). The interaction of AGEs with their receptor (RAGE) on VSMCs triggers intracellular oxidative stress by activating the transcription factor NF-κB (Yamagishi, 2011; San Martin et al., 2007). Experimental research has demonstrated that PCs therapy diminishes the proliferation and migration of human aortic smooth muscle cells (HASMCs) triggered by AGEs by elevating UCH-L1 protein levels, reducing IκB-α degradation, and curtailing NF-κB nuclear translocation in a dose-responsive manner (Qian et al., 2011). GSK-3β, a pro-apoptotic kinase, is crucial in managing cell survival and apoptosis. Overexpression of GSK-3β increases cellular susceptibility to apoptosis, while inhibition of GSK-3β diminishes the release of cytochrome c from mitochondria (Ryu et al., 2021). Li et al. (2011) showed that low concentrations of PCs (<12.5 mmol/L) enhance cell viability and proliferation in HUVECs, while higher concentrations (>25.00 mmol/L) induce apoptosis. This effect is attributed to PCs dose-dependently enhancing GSK-3β phosphorylation, attenuating caspase-3 activation, and inhibiting lactic acid adhesion protein expression, thereby mitigating oxidative stress and apoptosis induced by AGEs in HUVECs. These protective mechanisms lead to decreased intracellular ROS production and reduced apoptotic damage.

Atherosclerosis is a key marker of diabetic cardiovascular lesions. Oxidative stress, induced by hyperglycemia, plays a significant role in advancing atherosclerosis (Chen et al., 2011). In individuals with diabetes, ROS facilitate atherosclerosis by damaging endothelial cells while promoting proliferation, movement, and phenotypic changes in smooth muscle cells (Jay et al., 2006). MFG-E8, also known as lactoadhesin, adversely affects diabetic vascular disease by playing a role in oxidative stress and inflammatory responses (Silvestre et al., 2005). Prior studies have demonstrated that elevated glucose levels enhance ROS production by increasing MFG-E8 expression in adipose tissue (Aoki et al., 2007). Within the db/db mouse model, PCs effectively reduced VSMC proliferation and endothelial cell damage by decreasing MCP-1 and AGEs in mouse serum. Additionally, it reduced oxidative stress in vascular endothelial cells and decelerated atherosclerosis progression by enhancing aortic glutathione S-transferase theta 1 (GSTT1) activity. The suggested mechanism includes PCs impacting MFG-E8 expression, which subsequently mitigates atherosclerosis in db/db mice by reducing the secretion of chemokines involved in inflammation and oxidative stress via the pathways of ERK1/2 and MCP-1 (Yu et al., 2012) (Figure 11). Clinical studies have found (Peter et al., 2013) that the combined intake of isoflavones and flavan-3-ols improves vascular function and arterial stiffness in postmenopausal patients with type 2 diabetes, thereby slowing disease progression.

In conclusion, PCs improve diabetes-induced cardiovascular diseases through various mechanisms, particularly by regulating endothelial function, reducing oxidative stress, inhibiting inflammation, and modulating vascular smooth muscle cell proliferation, exhibiting significant neuroprotective and cardiovascular protective effects. PCs can slow the formation of atherosclerosis and vascular endothelial cell damage by regulating multiple signaling pathways, including AGEs, MFG-E8, and GSK-3β. Although these studies support the application of PCs in diabetes-related cardiovascular diseases, further clinical trials and validation are required to promote their clinical application.

#### 5.5.3 PCs and diabetic cardiomyopathy

Diabetic cardiomyopathy (DC) presents as abnormal cardiac structure and function in individuals with diabetes, occurring in the absence of significant coronary artery disease, hypertension, or valvular heart conditions (Jia et al., 2018; Dillmann, 2019). Recent research indicates that endothelial dysfunction is a key factor in the advancement of diabetic cardiomyopathy (Knapp et al., 2019). Several factors, including hyperglycemia, hyperinsulinemia, lipid metabolism imbalances due to insulin resistance, mitochondrial dysfunction, decreased NO activity, elevated ROS, and inflammatory dysregulation, adversely affect endothelial function (Moran et al., 2022). This dysfunction can lead to cardiac fibrosis and structural changes through disrupted myocardial metabolism. The involved processes include improper handling of intracellular Ca^2^⁺, ER stress, mitochondrial dysfunction, the accumulation of AGEs, and the buildup of the extracellular matrix (ECM). These alterations can ultimately lead to both diastolic and systolic cardiac dysfunction, potentially resulting in heart failure (Moran et al., 2022). Quantitative proteomic analysis using iTRAQ revealed that PCs reverse s100A11 protein expression, a component in the S100 calcium-binding protein family involved in cellular regulation, and lower serum AGE levels in db/db mice. The hypothesis suggests that PCs activate the S100A11/RAGE/PPARγ signaling pathway, resulting in reduced RAGE expression and alleviation of cardiac fibrosis triggered by AGEs in type 2 diabetic rats (Luan et al., 2014). Mitochondria act as the primary sources of cellular energy and also produce ROS. A notable correlation exists between ROS production and the deterioration of cardiac function and structure (Bou-Teen et al., 2021). Dysfunctional mitochondrial activity due to hyperglycemia serves as an early sign of endothelial dysfunction, hastening the advancement of cardiovascular disease (Yuan et al., 2019). Normal mitochondrial function reduces myocardial oxidative damage, while abnormal mitochondrial function can cause chronic myocardial hypoxia and exacerbate myocardial fibrotic degeneration by impairing respiratory function. PCs stimulate myocardial mitochondrial phosphorylation, modulating mitochondrial uncoupling rates and affecting respiratory function (Kopustinskiene et al., 2015).

In conclusion, PCs can alleviate diabetes-induced myocardial hypertrophy, cardiac fibrosis, and heart failure, demonstrating potential cardioprotective effects by regulating the S100A11/RAGE/PPARγ signaling pathway, improving mitochondrial function, reducing oxidative stress, and inhibiting the formation of AGEs.

## 6 Advantages, limitations, and future directions

As a potent antioxidant, PCs exhibit significant therapeutic potential in the treatment of diabetes and its complications through their anti-inflammatory, antioxidant, and anti-glycation properties. These plant metabolites improve endothelial function, reduce insulin resistance, and protect pancreatic β-cell function, offering a multidimensional strategy for diabetes treatment. However, current studies face several limitations. Most studies are restricted to *in vitro* experiments or animal models, lacking clinical trials in humans, particularly long-term efficacy and safety evaluations. Additionally, the low bioavailability of PCs, primarily due to poor gastrointestinal absorption, poses challenges for clinical application. The precise mechanisms of PCs remain unclear, necessitating further investigation into their molecular pathways.

Future research should focus on enhancing the bioavailability and pharmacokinetics of PCs, potentially through novel delivery systems like nanoparticles or their combination with other plant bioactive metabolites, to optimize therapeutic outcomes. Large-scale randomized controlled trials are essential to validate their efficacy in patients with diabetic complications, particularly for clinical assessments of diabetic retinopathy, nephropathy, and neuropathy. Additionally, investigating the synergistic effects of PCs with other natural compounds or conventional drugs may unveil more comprehensive therapeutic strategies. More importantly, greater emphasis should be placed on studying their molecular mechanisms, especially their effects on inflammatory pathways and oxidative stress, to develop more targeted therapeutic approaches. Personalized medicine approaches, considering individual genetic and metabolic variations, may further optimize the clinical application of PCs in diabetes and its complications.

## 7 Prospectives and conclusion

PCs are versatile bioactive compounds with significant potential in the prevention and management of diabetes and its related complications. They not only regulate glucose metabolism but also exhibit strong antioxidant and anti-inflammatory properties, which enhance insulin sensitivity and improve endothelial function. Experimental studies have shown that PCs offer protective effects against diabetic nephropathy, neuropathy, and cardiovascular diseases. Despite these promising findings, most research has been limited to *in vitro* and animal models, highlighting the need for clinical validation.

Future research should prioritize comprehensive clinical trials to confirm the efficacy and safety of PCs in human subjects. Investigating synergistic interactions between PCs and existing antidiabetic therapies could lead to more effective treatment protocols with minimized side effects. Furthermore, advancements in delivery systems and the development of standardized formulations are crucial for enhancing the bioavailability and therapeutic effectiveness of PCs. Establishing uniform criteria for PC purity, composition, and dosage will not only ensure reproducible and effective results but also streamline clinical trials and future regulatory approval processes.

In summary, PCs present a promising adjunctive option for diabetes management, contributing to better glycemic control and the mitigation of complications. Continued research and clinical investigations are essential to fully realize the therapeutic benefits of PCs, ultimately aiming to improve long-term outcomes and the quality of life for individuals with diabetes.

## Abbreviations

PCs, Procyanidins; DM, Diabetes Mellitus; T1DM, Type 1 Diabetes Mellitus; T2DM, Type 2 Diabetes Mellitus; DN, Diabetic Nephropathy; DR, Diabetic Retinopathy; DN, Diabetic Neuropathy; CVD, Cardiovascular Disease; DFU, Diabetic Foot Ulcers; AGE, Advanced Glycation End-products; RAGE, Receptor for Advanced Glycation End-products; VEGF, Vascular Endothelial Growth Factor; DCD, Diabetic Cardiomyopathy; GSH-Px, Glutathione Peroxidase; NQO1, NAD(P)H Quinone Dehydrogenase 1; ZO-1, Zonula Occludens-1; TLR4, Toll-like Receptor 4; MCP-1, Monocyte Chemoattractant Protein-1; AMPK, AMP-activated Protein Kinase; GSK-3β, Glycogen Synthase Kinase 3 Beta; ERK1/2, Extracellular Signal-Regulated Kinases 1/2; ACC, Acetyl-CoA Carboxylase; GLUT4, glucose transporter type 4; ATGL, Adipose Triglyceride Lipase; CPT1A, Carnitine Palmitoyltransferase 1A; SIRT1, Sirtuin 1; NRF1, Nuclear Respiratory Factor 1; PERK, Protein kinase RNA-like ER kinase; IRE1, Inositol-Requiring Enzyme 1; ATF6, Activating Transcription Factor 6; ROS, Reactive Oxygen Species; TGF-β, Transforming Growth Factor Beta; EMT, Epithelial-to-Mesenchymal Transition; MAPK, Mitogen-Activated Protein Kinase; mTOR, Mechanistic Target of Rapamycin; NF-κB, nuclear factor kappa-B; miR-34a, MicroRNA-34a; Keap1, Kelch-like ECH-associated protein 1; ARE, Antioxidant Response Element; 3-NT, 3-Nitrotyrosine; 4-HNE, 4-Hydroxynonenal; PI3K, Phosphoinositide 3-Kinase; CAT, Catalase; OXR1, Oxidation Resistance 1; NRF2, Nuclear Factor Erythroid 2-Related Factor 2; JAK, Janus Kinases; SOD, Superoxide Dismutase; RNS, Reactive Nitrogen Species; M1/M2, Macrophage phenotypes 1 and 2; IL-6, Interleukin 6; IL-17, Interleukin 17; IL-12, Interleukin 12; IL-1β, Interleukin 1 Beta; Arg1, Arginase 1; Ym1, Chitinase-Like Protein 3; Fizz1, Found in Inflammatory Zone 1; ABCG1, ATP Binding Cassette Subfamily G Member 1; CD36, Cluster of Differentiation 36.
